# Contributions of Protein-Coding and Regulatory Change to Adaptive Molecular Evolution in Murid Rodents

**DOI:** 10.1371/journal.pgen.1003995

**Published:** 2013-12-05

**Authors:** Daniel L. Halligan, Athanasios Kousathanas, Rob W. Ness, Bettina Harr, Lél Eöry, Thomas M. Keane, David J. Adams, Peter D. Keightley

**Affiliations:** 1 Institute of Evolutionary Biology, University of Edinburgh, Edinburgh, United Kingdom; 2 Max-Planck Institute for Evolutionary Biology, Plön, Germany; 3 The Roslin Institute and R(D)SVS, University of Edinburgh, Midlothian, United Kingdom; 4 The Wellcome Trust Sanger Institute, Hinxton, Cambridge, United Kingdom; University of Wisconsin–Madison, United States of America

## Abstract

The contribution of regulatory versus protein change to adaptive evolution has long been controversial. In principle, the rate and strength of adaptation within functional genetic elements can be quantified on the basis of an excess of nucleotide substitutions between species compared to the neutral expectation or from effects of recent substitutions on nucleotide diversity at linked sites. Here, we infer the nature of selective forces acting in proteins, their UTRs and conserved noncoding elements (CNEs) using genome-wide patterns of diversity in wild house mice and divergence to related species. By applying an extension of the McDonald-Kreitman test, we infer that adaptive substitutions are widespread in protein-coding genes, UTRs and CNEs, and we estimate that there are at least four times as many adaptive substitutions in CNEs and UTRs as in proteins. We observe pronounced reductions in mean diversity around nonsynonymous sites (whether or not they have experienced a recent substitution). This can be explained by selection on multiple, linked CNEs and exons. We also observe substantial dips in mean diversity (after controlling for divergence) around protein-coding exons and CNEs, which can also be explained by the combined effects of many linked exons and CNEs. A model of background selection (BGS) can adequately explain the reduction in mean diversity observed around CNEs. However, BGS fails to explain the wide reductions in mean diversity surrounding exons (encompassing ∼100 Kb, on average), implying that there is a substantial role for adaptation within exons or closely linked sites. The wide dips in diversity around exons, which are hard to explain by BGS, suggest that the fitness effects of adaptive amino acid substitutions could be substantially larger than substitutions in CNEs. We conclude that although there appear to be many more adaptive noncoding changes, substitutions in proteins may dominate phenotypic evolution.

## Author Summary

We present an analysis of the genome sequences of multiple wild house mice. Wild house mice are about ten times more genetically diverse than humans, reflecting the large effective population size of the species. This manifests itself as more effective natural selection acting against deleterious mutations and favouring advantageous mutations in mice than in humans. We show that there are strong signals of adaptive evolution at many sites in the genome. We estimate that 80% of adaptive changes in the genome are in gene regulatory elements and only 20% are in protein-coding genes. We find that nucleotide diversity is markedly reduced close to gene regulatory elements and protein-coding gene sequences. The reductions around regulatory elements can be explained by selection purging deleterious mutations that occur in the elements themselves, but this process only partially explains the diversity reductions around protein-coding genes. Recurrent adaptive evolution, which can also cause local reductions in diversity via selective sweeps, may be necessary to fully explain the patterns in diversity that we observe surrounding genes. Although most adaptive molecular evolution appears to be regulatory, adaptive phenotypic change may principally be driven by structural change in proteins.

## Introduction

Only about 1% of the mammalian genome encodes proteins [1]. Purifying selection is apparent in protein-coding sequences, where both diversity within species and divergence between species is markedly reduced. Additionally, selection prevents deleterious mutations in protein-coding genes from rising in frequency, which creates an excess of rare variants. In some mammalian species, there is also evidence of widespread adaptive evolution in proteins, manifest as an excess of substitutions compared to that expected under a neutral model [2]–[4]. However, little is known about selection operating on regulatory sequences present in the noncoding fraction of the genome or about the strength of selection operating on adaptive substitutions in general. Studies of interspecific divergence of noncoding DNA sequences among mammalian species imply that only ∼5% of the noncoding genome is conserved, and therefore is likely to be subject to purifying selection [5]. Conversely, a high proportion (as much as 80%) of the genome has been classified as “functional” by virtue of displaying reproducible biochemical signatures [6], but the evolutionary significance of this has been challenged [7]–[8]. The overall evolutionary rate and fitness change associated with advantageous and deleterious mutations in noncoding DNA are essentially unknown.

The question of the relative contributions of protein-coding versus noncoding DNA to genetic variation for fitness and adaptive evolution is not new. King and Wilson [9] argued that changes in proteins (also known as structural changes) are unlikely to explain the myriad phenotypic adaptations that distinguish humans and chimpanzees, and proposed that regulatory change in noncoding sequences affecting the timing and tissue-specificity of gene expression must therefore dominate adaptive evolutionary change. There is empirical evidence from genetic mapping experiments both supporting [10]–[12] and contradicting this view [13]. On theoretical grounds, it has been argued that regulatory change should cause fewer harmful pleiotropic effects than mutations affecting proteins [10], and there is evidence in *Drosophila* and mammals from patterns of diversity within and between species suggesting the presence of positive selection in noncoding DNA [14]–[17].

Here, we study genome-wide nucleotide diversity within the house mouse subspecies *M. m. castaneus* and nucleotide divergence from two outgroup species, *M. famulus*, and *Rattus norvegicus* (the brown or Norway rat). Genome sequences of wild mice were obtained from individuals sampled from their ancestral range in NW India [18]; see Materials and Methods. The effective population size of house mice from this region has been estimated to be nearly 10^6^ [2]. Nucleotide diversity levels approach those seen in some invertebrates and some signals of natural selection are stronger than seen in humans, making wild house mice a powerful system for studying natural selection in the mammalian genome [16]–[18].

We use these data firstly to estimate and compare the distributions of fitness effects (DFEs) for deleterious mutations occurring in protein-coding genes and conserved noncoding elements (CNEs), many of which have functions regulating gene expression. Secondly, we attempt to quantify the relative numbers of adaptive coding and noncoding substitutions based on nucleotide divergence between *M. m. castaneus* and the two outgroup species. However, it is important to consider not only the rate, but also the fitness effects of adaptive substitutions, since a category of sites showing a low rate of adaptive substitution could make a larger contribution to adaptation if the substitutions tend to have stronger effects on fitness. The relative contributions of structural and regulatory adaptive fitness change has not previously been explored in any depth. To address this issue, we investigate patterns of nucleotide diversity surrounding protein-coding exons and CNEs. As a consequence of genetic linkage, diversity is expected to be reduced close to sites where advantageous mutations regularly spread to fixation (causing selective sweeps), but may also be reduced as a consequence of purging of deleterious mutations (background selection). We attempt to quantify the relative impact of these two forces on diversity at sites closely linked to exons and CNEs.

## Results

We investigated genome-wide patterns of nucleotide diversity in ten wild house mice sampled from their ancestral range in NW India [18]. Individuals were sequenced on the Illumina GAIIx and HiSeq platform to a mean depth of ∼30-fold, allowing accurate inference of genotypes at the vast majority of sites in the genome (Text S1), and genotype calls appear to be more accurate than calls made previously by Sanger sequencing of selected loci (Text S1). We also sequenced the genome of a related mouse species, *M. famulus*, at a mean depth of 27-fold and used this as an alternative outgroup to the brown rat (*Rattus norvegicus*) (Text S1).

The focus of our analysis was the annotated set of murid protein-coding genes, their untranslated regions (UTRs) and a set of mammalian conserved noncoding elements (CNEs), many of which have roles regulating gene expression, especially in development [19], [20]. We identified 1.51×10^6^ noncoding elements (CNEs) in the mouse genome using phastCons [21], based on data from placental mammals, whilst excluding information from mouse and rat in order to avoid an ascertainment bias in divergence down the mouse and rat lineages (Text S1). This is equivalent to approximately four times the number of nonsynonymous sites in the genome (Table 1) and is consistent with estimates of the amount of conserved mammalian noncoding DNA [5].

**Table 1:** Results from DFE-alpha.

Parameter	Zero-fold	Two-fold	UTRs	CNEs
***n_t_ (Mb)***	23.0	2.79	4.24	82.1
***N_e_s***	9.5×10^5^	→∞	250	45
	[2.6×10^5^, 3.5×10^7^]	[4.0×10^9^, →∞]	[140, 500]	[40], [50]
***β***	0.11	→0	0.050	0.16
	[0.088, 0.13]	[→0, 0.066]	[0.050, 0.050]	[0.15, 0.17]
***u_n_***	0.16	0.20	0.65	0.40
	[0.16, 0.17]	[0.19, 0.20]	[0.63, 0.67]	[0.39, 0.41]
**0<** ***N_e_s*** **<1**	0.17	0.21	0.65	0.44
	[017, 0.18]	[0.20, 0.21]	[0.65, 0.69]	[0.43, 0.44]
**1<** ***N_e_s*** **<10**	0.052	0.025	0.082	0.19
	[0.041, 0.057]	[0.024, 0.033]	[0.079, 0.084]	[0.18, 0.20]
***N_e_s*** **>10**	0.77	0.77	0.25	0.37
	[0.77, 0.78]	[0.76, 0.78]	[0.22, 0.27]	[0.37, 0.37]
***u_n_***	0.16	0.20	0.65	0.40
	[0.16, 0.17]	[0.19, 0.20]	[0.63, 0.67]	[0.39, 0.41]
***α***	0.32	0.38	0.19	0.19
	[0.28, 0.35]	[0.36, 0.42]	[0.16, 0.23]	[0.18, 0.21]
***ω_a_***	0.077	0.12	0.15	0.097
	[0.067, 0.087]	[0.12, 0.14]	[0.12, 0.19]	[0.091, 0.10]
***n_a_ (Mb)***	0.32	0.063	0.12	1.4
	[0.28, 0.36]	[0.059, 0.070]	[0.096, 0.15]	[1.4, 1.5]

*n_t_* is the total number of sites in the reference genome corresponding to each mutually exclusive site class (including non-canonical spliceforms in the case of protein-coding exons). *N_e_s* (the product of the mean homozygous effect of a deleterious mutation and the effective population size) and *β* (the gamma shape parameter, which has a lower estimable value of 0.05 within DFE-alpha) are the inferred parameters of the DFE, from which we calculate the mean fixation probability of a deleterious mutation relative to a neutral mutation (*u_n_*) and estimates of the proportion of deleterious mutations in three ranges of fitness effects (on a scale of *N_e_s* = 0–1, 1–10 and 10+). From estimates of divergence from rat at selected and neutral sites, we calculate estimates of the proportion of adaptive substitutions (*α*) and the rate of adaptive substitution relative to the rate of synonymous substitution (*ω_a_*) (results are shown for non-CpG-prone sites). *n_a_* is an estimate of the total number of adaptive substitutions between mouse and rat attributable to each site class and is calculated from *n_a_* = *ω_a_ n_t_ d_s_*, where *d_s_* = 0.18, an estimate of divergence for synonymous sites. 95% confidence limits are shown in square brackets.

**Figure 1:**
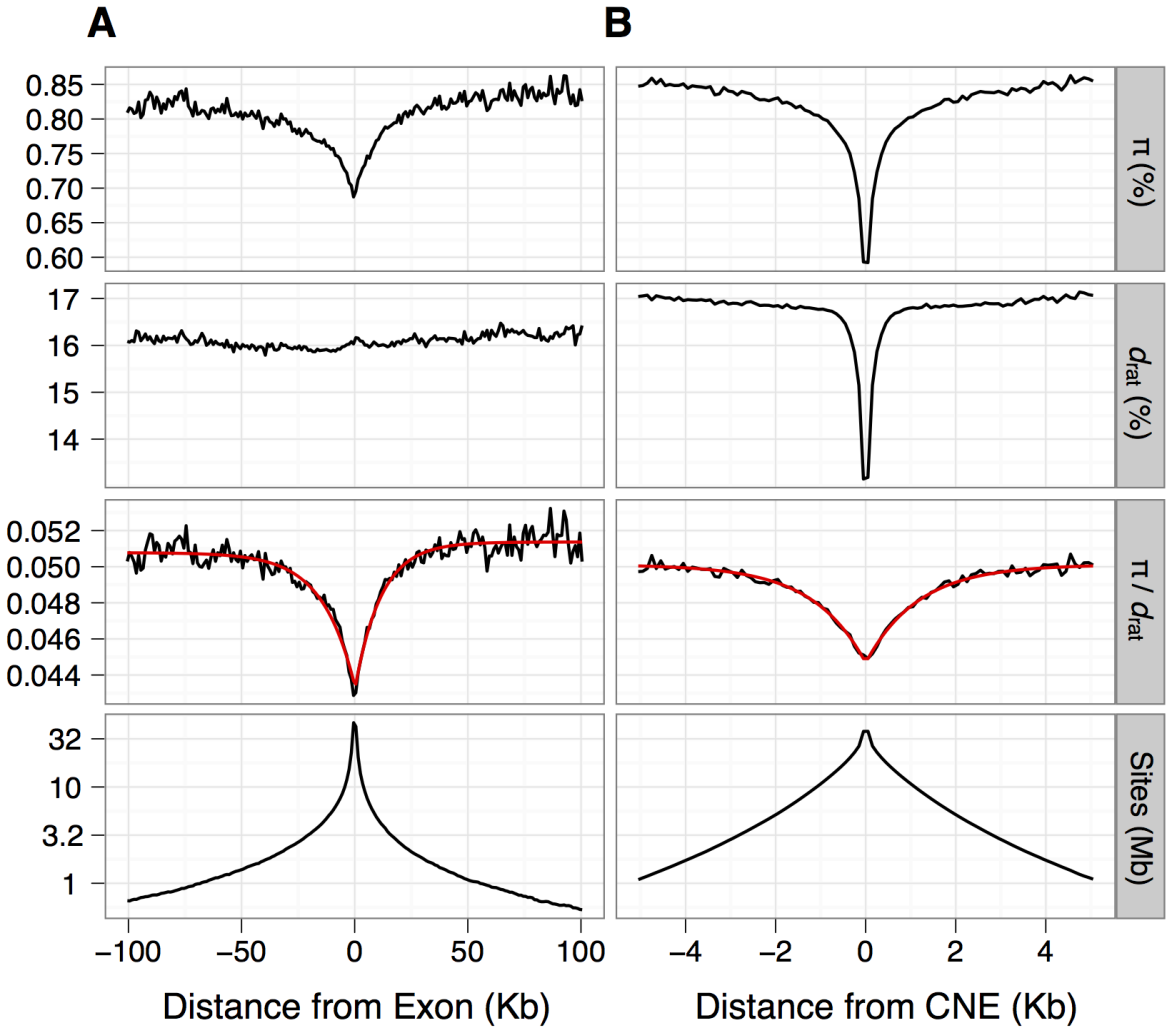
Estimates of mean nucleotide diversity (*π*) in house mice, divergence to rat (*d_rat_*) and their ratio (*π*/*d*) plotted against the distance from the nearest protein-coding exon (panel A) or CNE (panel B). Mean estimates of *π*/*d* can be approximated well by a negative exponential function (red line), obtained by fitting the function f(*x*) = *A*(1-*B*(exp(-*x/d*))) to mean *π*/*d* by nonlinear least squares (see Materials and Methods for details). The bottom panel shows the number of sites (in Mb) on a log scale that contribute to each bin.

For inferences of selection, we required putatively neutrally evolving sequences that are tightly linked to the functional sequences [22]. For nonsynonymous sites and UTRs, we used synonymous sites as a neutral reference. In mammals, however, there is evidence that some synonymous sites are under weak selection [23], [24], although purifying selection appears to be substantially weaker in murids than in primates [25]. Within the MK framework, selection on synonymous sites could upwardly bias estimates of the rate of adaptive evolution if selection reduced synonymous nucleotide divergence proportionally more than diversity. To investigate the extent of bias, we compared the results from assuming synonymous sites as the neutral reference to results based on assuming ancestral repeats (ARs) in introns as the neutral reference, under the assumption that ARs are a good proxy for neutrally evolving sites (e.g., [26]). For the neutral reference of CNEs, we used sections of the genome close to CNEs (masked for any protein-coding exons, UTRs or other CNEs) that are located far enough away from the CNEs such that mean divergence from rat approximates that of neutrally evolving ancestral repeats (see Methods). We found that mean nucleotide divergence approximates that of ancestral repeats at sites located more than ∼500 bp away from the nearest CNE (Figure 1).

### Genome-wide nucleotide diversity in wild house mice and divergence from the rat

**Figure 2:**
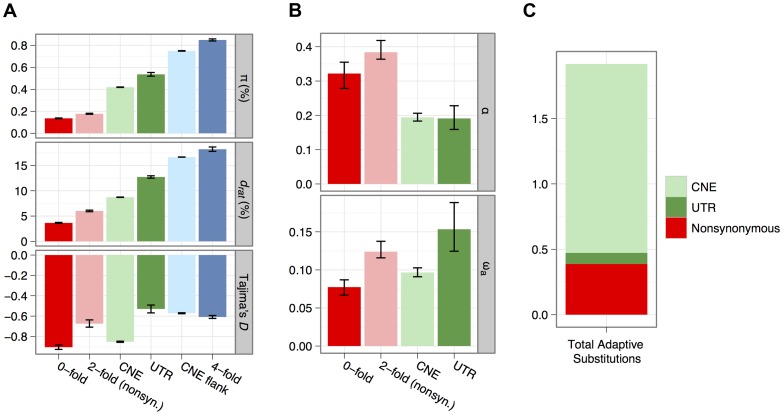
Estimates of diversity (*π*), divergence to rat (*d_rat_*) and Tajima's *D* for all categories of sites analysed for non-CpG-prone sites (A). Estimates of *α* and *ω_a_* for each category of selected site, obtained from an analysis of the site frequency spectrum using non-CpG-prone sites and divergence to rat (B). Inferred numbers of adaptive substitutions separating mouse and rat for the mutually-exclusive selected sequence categories, assuming there are on average 0.18 substitutions per neutral site between mouse and rat, estimated from four-fold degenerate sites (C).

We calculated mean nucleotide diversity (*π*) and mean divergence (*d*) from the brown rat for nonsynonymous sites, synonymous sites and UTRs of protein-coding genes and CNEs and the flanks of CNEs (Figure 2A). Diversity of synonymous sites and CNE flanks approaches 1%, consistent with previous surveys of protein-coding genes [2] and CNEs [17]. Diversity is therefore ∼10-fold higher than in humans and higher than in other wild house mouse populations [3], [18], [27]. As expected, mean nucleotide divergence between mouse and rat and diversity within *M. m. castaneus* at CNEs and nonsynonymous sites are much lower than the corresponding values for synonymous sites and CNE flanks (Figure 2A), consistent with net purifying selection. This is corroborated by an excess of rare variants and a more negative Tajima's *D* than at synonymous sites (Figure 2A).

### Distributions of fitness effects of deleterious mutations

We obtained the folded site frequency spectrum (SFS = the distribution of minor allele frequencies across sites) for *M. m. castaneus* from each of our focal classes of sites (nonsynonymous sites, UTRs and CNEs) and for tightly linked, putatively neutral sites. We then used these SFSs to estimate the distribution of fitness effects (DFE) of deleterious mutations in the focal classes (Table 1, S1, S2; Figure S1) using the program DFE-alpha [28] (see Materials and Methods). To do this, we first fit a demographic model to the neutral SFS by maximum likelihood with a step change in population size. Then, assuming the estimated demographic parameters from this model (Table S1), we fit a gamma DFE of new deleterious mutations to the selected SFS. This model makes the assumption that advantageous mutations are sufficiently rare as to make a negligible contribution to polymorphism.

The inferred gamma DFEs for each selected site class are highly leptokurtic (Table 1), i.e, estimates of the shape parameter of the distribution, *β*, are all less than 0.19. This implies that most nonsynonymous mutations are strongly deleterious (we infer that 77% have fitness effects greater than *N_e_s* = 10), and that there are relatively few nearly neutral nonsynonymous mutations (∼20% have fitness effects less than *N_e_s* = 1) (Table 1). In particular, this estimated proportion of nearly neutral amino acid-changing mutations is markedly lower than estimates for human populations [28]–[30] which is likely to be a consequence of the larger recent effective population size of wild house mice. However, nearly neutral mutations in UTRs and CNEs are relatively abundant and strongly deleterious mutations are less frequent (we estimate that ∼65% and ∼44% of mutations have fitness effects less than *N_e_s* = 1, whereas ∼25% and ∼37% have fitness effects greater than *N_e_s* = 10, for UTRs and CNEs respectively). If the DFE is highly leptokurtic (as in these cases), the mean effect of a deleterious mutation is highly sensitive to the frequency of strongly deleterious mutations that are essentially absent from the data, and cannot be accurately estimated. However, the estimated proportions of mutations with selective effects in different ranges in more robust to the model assumptions [28], [31].

### Rates of adaptive substitution in protein-coding genes and CNEs

We used DFE-alpha, incorporating an extension of the McDonald-Kreitman test [32], [33], to estimate the proportion of nucleotide differences (*α*) in protein-coding genes, UTRs and CNEs that were driven to fixation by positive selection (Materials and Methods). To do this, we calculate the mean fixation probability of a deleterious mutation relative to a neutral mutation (*u_n_*) from the inferred DFE (Table 1), and use this to estimate the expected number of fixed differences between mouse and rat attributable to neutral and slightly deleterious mutations [32]. An excess of observed substitutions compared to that expected can be quantified as *α*, the fraction of adaptive substitutions, or *ω_a_*, the rate of adaptive substitution relative to the rate of neutral substitution (*d_s_*, Materials and Methods). We obtained high estimates of *α* and *ω_a_* for all site classes, indicating that there has been widespread adaptive evolution in proteins, UTRs and CNEs (Table 1, Fig. 1B, Table S2).

In order to check the robustness of our estimates of the DFE and *α* and *ω_a_*, we investigated alternative neutral reference classes of sites and fitted a more complex demographic model to the neutral site data. Similar results are obtained if we assume AR sites within introns as a neutral reference (Table S3). We then investigate an alternative demographic model incorporating two step changes in the population size. This gave similar fits to the neutral SFSs as the single step change model (Table S4). Our results were not substantially affected by the choice of the neutral reference for CNEs, and we obtained similar results for CNEs located near to and far from exons (Table S5). Previous work suggest that estimates of the DFE and the rate of adaptive evolution from DFE-alpha are substantially robust to mis-specification of the demographic model [28], [32], as long as the estimated demographic parameters provide a good model fit to the observed folded neutral SFS. For similar reasons, estimates of the rate of adaptive evolution are robust to the presence of genetic linkage, although this can lead to misinference of the demographic parameters [34]. We investigated the extent to which our estimates of *α* and *ω_a_* depend on our assumption that the DFE is gamma distributed by fitting a second model of the DFE in which selective effects are divided into three discrete bins [31]. In this model we allow three bins of mutation effects at *s* = 0 (i.e. neutral), *s* = *s_2_* (estimated) and *s* = 1 (i.e., lethal) and estimate the relative proportion of mutations with these effects (*p_1_*, *p_2_* and *p_3_*; Table S6). Our estimates of *α* and *ω_a_* depend on *u_n_*, which is obtained from the DFE (Materials and Methods). For both the gamma model and the discrete effects model, our estimates of *u_n_* are similar, although the resulting estimates of *α* and *ω_a_* are somewhat lower when using the discrete model (Table 1, S6). The results are also robust to the choice of outgroup (Table S2; see Text S1).

The total rate of adaptive substitution per generation (*n_a_*) attributable to a particular class of sites is proportional to its rate of adaptive substitution (*ω_a_*):
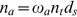
(1)where *n_t_* is the total number of sites in that class (Table 1) and *d_s_* is the divergence for the neutral reference class of sites. A comparison of *n_a_* estimates among nonsynonymous sites, UTRs and CNEs therefore suggests that the majority (>70%) of adaptive nucleotide substitutions in the murid genome occur in CNEs and that only about 20% occur at nonsynonymous sites in protein-coding genes (Table 1; Figure 2C).

### Nucleotide diversity around amino acid substitutions

**Figure 3:**
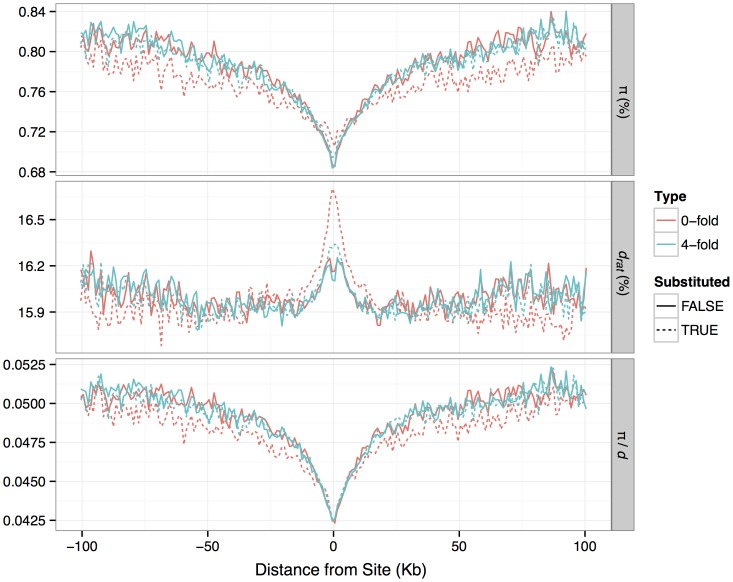
Patterns of nucleotide diversity (*π*), divergence to rat (*d*) and *π/d*, in the flanks of zero-fold degenerate and four-fold degenerate protein-coding sites identified as either having a fixed substitution between *M. m. castaneus* and *M. famulus* or no substitution.

It has been suggested that the pattern of nucleotide diversity around selected sites can provide information about the rate and selective effects of adaptive mutations [35], [36]. Diversity is expected to be reduced at sites partially linked to an adaptive substitution, so we would expect to observe reductions in diversity around substituted sites if some fraction of substitutions were driven by positive selection. In our data we see substantial reductions in mean *π* in the regions flanking nonsynonymous sites that have experienced a nucleotide substitution between *M. m. castaneus* and the closely related *M. famulus* (Figure 3). We also see slightly increased *d* (mouse-rat divergence) near nonsynonymous substituted sites, indicative of locally correlated rates of nonsynonymous substitution, which may reflect local variation in constraint or mutation rate. Notably, we still see reductions in mean *π* when controlling for *d*. However, the reduction in mean *π*/*d* around substituted nonsynonymous sites is very similar to that around substituted synonymous sites, as observed by Hernandez *et al.* [36] (Figure 3; Text S1). Furthermore, a similar drop in mean *π*/*d* is also observed around nonsynonymous sites that have not experienced a substitution.

As discussed below, these patterns can be explained by the presence of many linked selected sites jointly contributing to reductions of *π*/*d*, obscuring the contributions of individual substitutions, and/or effects of background selection. Thus, there appears to be limited information present in measures of mean *π*/*d* in flanking regions to infer the strength of positive selection at substituted sites, at least in wild house mice and hominids, unless the effects of selection on linked sites are taken into account.

### Nucleotide diversity around exons and CNEs

Information about the frequency and strength of selected mutations may also be contained in the pattern of nucleotide diversity and divergence surrounding functional genomic elements, such as exons or CNEs [37]. Both diversity and divergence are expected to be influenced to similar extents by variation in the mutation rate or negative selection acting in the flanking sequences. However, diversity is also expected to be influenced by selection at linked sites (either in the form of hitchhiking or background selection). Thus, it is possible in principle to tease apart the contribution of linked selection to diversity by controlling for divergence. We see reductions in diversity and divergence in the flanks of CNEs, indicative of direct negative selection, consistent with a previous study [17], and indicating that our defined CNEs may not include the entire underlying functional elements (Figure 1). However, we observe substantial reductions in *π*/*d*, around both exons and CNEs, suggesting that there is an effect of positive or negative selection in exons and CNEs on diversity in the linked sequences flanking them [36], [38], [39]. Notably, mean *π*/*d* in the flanks of both exons and CNEs is well approximated by a negative exponential function, and the fit of this model implies that *π*/*d* is reduced over a ∼10-fold wider area around exons than around CNEs (Figure 1). We obtained similar results if sequences that are most likely to experience direct negative selection are excluded from exon and CNE flanks (Text S1 and Table S7), supporting the conclusion that the observed reductions reflect the action of selection on the linked functional elements.

When interpreting these patterns, it is important to account for the spatial and length distribution of selected elements in the genome. In particular reductions in *π*/*d* (Figure 1) will be influenced by the following factors: 1. Selection on exons can contribute to the diversity reductions observed in the flanks of CNEs and *vice versa* (i.e., the panels in Figure 1 are not independent from each other). 2: CNEs and exons vary dramatically in length. 3: Both CNEs and exons are clustered (Figure S2), such that more than 80% of exons lie within 10 Kb of another exon and more than 75% of CNEs are within 1 Kb of another CNE. 4: Exons tend to cluster with CNEs (Figures S3). This implies that reductions in diversity around exons and CNEs (which extend over ∼100 Kb and ∼10 Kb, respectively) are likely to be affected by selection, not only on the nearest exon or CNE, but many other closely linked CNEs and exons as well.

To address these issues, we attempted to model *π*/*d* calculated within 200 bp or 1 Kb non-overlapping windows throughout the genome (at non-exonic and non-CNE sites). Although we attempted to fit a variety of simpler models (Text S1), the best fitting model (model C, as measured by *r^2^*) was one where we considered the effects of all linked selected sites on *π*/*d*, rather than just the effect of the nearest exon and CNE (Table S8). In this model, we reasoned that *π*/*d* for any given non-overlapping window depends on the neutral (unreduced) level of *π*/*d* and the combined reductions attributable to all linked selected bases (in exons or CNEs). We assumed that these diversity reductions decay exponentially around each selected base within an exon or CNE and that the individual reductions combine multiplicatively to give the total predicted reduction (see Materials and Methods). We estimated two exponential rate parameters, for exonic and CNE sites, simultaneously.

**Figure 4:**
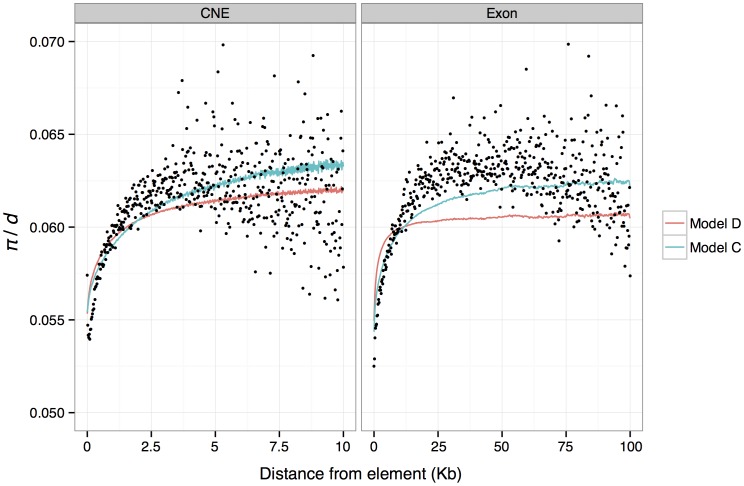
Mean predicted and observed *π/d* in genomic windows binned by absolute distance from exon and CNE boundaries. Mean observed *π/d* are shown as black dots. Mean predicted values from a model assuming that reductions in diversity for each linked selected site are exponentially distributed (model C) are shown as blue lines. Mean predictions from the best fitting model of background selection (Model D) are shown as red lines.

The results from this model imply that a single exonic or CNE site reduces diversity at a linked neutral site by 0.0013% and 0.019%, respectively. Therefore, in the absence of any influence from other linked selected exons or CNEs, we predict a reduction in *π*/*d* of only 0.23% and 1%, for an average exon (176 bp) or CNE (54 bp), respectively. Note that these predicted reductions, are much smaller than the observed average reductions of 16% and 14% around exons and CNEs respectively (Figure 1). The discrepancy can be explained by the contribution of multiple selected elements on the observed reductions in *π*/*d*. To further investigate this finding, we obtained *π*/*d* predictions from model C for each genomic window, binned these values according to distance from the nearest exon/CNE, and plotted the average for each bin against distance (Figure 4). These predictions give a good fit to the observed data. This model also implies that reductions in diversity attributable to a single exonic base are ∼5× wider on average than those attributable to a single conserved noncoding base (parameters *p_3_* and *p_5_* for model C in Table S8).

### Background selection

Our results suggest that the substantial reductions in diversity around CNEs and exons could be explained by the cumulative effects of many linked selected sites. Can these reductions in neutral diversity be explained solely as a result of deleterious mutation? To investigate this, we attempted to model background selection (BGS) [40], a process whereby reductions in diversity result from selection against deleterious mutations at linked sites [41]. We used the approach and software described by McVicker et al. [39] to predict reductions in diversity throughout the genome as a result of deleterious mutations occurring within exons and CNEs. In this model we assume a mutation rate of 3.79×10^−9^, and a recombination rate of 0.528 cM/Mb [42]. We also assume that reductions from each element type (exons and CNEs) combine multiplicatively and that the effects of deleterious mutations in CNEs and exons follow an exponential distribution, the parameters of which we estimate by least-squares (Materials and Methods). We subsequently tested the fit of a gamma distribution of deleterious mutation effects with a range of shape parameters (0.25, 0.75 and 2.0) and found that the fit to the data (as measured by the *r* ^2^) was very similar across the range of shape parameters tested (data not shown).

The best-fitting BGS model suggests that deleterious mutations at nonsynonymous and CNE sites have absolute mean selection coefficients of 4×10^−5^ and 2×10^−5^, respectively (Table S8), implying mean scaled selection coefficients (*N_e_s*) of ∼44 and ∼22, respectively (assuming an effective population size in mice of 5.6×10^5^ and dominance coefficients of 0.5). BGS explains nearly as much variation (in terms of *r* ^2^) as model C described above. The results are also consistent in that they indicate that the substantial drops in mean diversity around exons and CNEs are caused by the cumulative effects of many selected elements, and that only small reductions are attributable to a single average length exon or CNE (i.e, the BGS model predicts a drop of ∼1% for both). We binned predictions of relative diversity according to distance from the nearest exon/CNE and plotted average for each bin against distance (Figure 4). Notably, although there is a good match between the average predicted diversity and the observed mean diversity in the flanks of CNEs, the width of the predicted reductions in diversity around exons fits the observations poorly. Note, however, that we also infer that there is substantial adaptive evolution in both CNEs and exons, so the observed diversity patterns are unlikely to be solely a consequence of BGS.

## Discussion

We generated deep whole-genome sequences of ten wild house mice of the subspecies *M. m. castaneus* from the species' ancestral range in NW India [18] and the genome of a single *M. famulus* individual. Confirming previous work, house mice from the NW Indian population are highly diverse which is likely to be a reflection of their large effective population size. We found clear evidence for adaptive evolution throughout the genome. We used these data to investigate the nature of the selective forces operating in coding and noncoding DNA. The principal findings from our work are as follows.

### Distributions of fitness effects of deleterious mutations

We used genome-wide polymorphism data to estimate the DFE of new deleterious mutations for non-synonymous sites, UTRs and non-coding elements conserved across mammals. The inferred DFEs are highly leptokurtic in all cases. Under a model of mutation-selection balance, the equilibrium genetic variance is proportional to the product of the mean effect of a deleterious mutation and the genomic mutation rate. We obtain much higher estimates of the mean effect of a nonsynonymous mutation than a CNE mutation (Table 1). This suggests that standing genetic variation for fitness could be dominated by variation in protein-coding genes. Precise partitioning of the variation is not possible, however, because estimates of the mean effect of a deleterious mutation depend on the frequencies of very rare, strongly selected alleles that are virtually absent from the data [43].

### Rates of adaptive molecular evolution

We calculated two measures of adaptation, the rate adaptive substitution relative to neutrality (*ω_a_*) and the fraction of substitutions that are adaptive (*α*). The method [32] attempts to account for the contribution of slightly deleterious mutations to polymorphism and divergence and the impact of recent demographic change. Consistent with previous work [2], [16], [17], estimates of *α* and *ω_a_* are quite high, suggesting that ∼30% and ∼20% of nonsynonymous and CNE/UTR substitutions, respectively, are driven to fixation by positive selection (Figure 2). These estimates disregard slightly advantageous mutations, but their contribution to the folded SFS is essentially indistinguishable from that of slightly deleterious mutations [43]. These values are considerably higher than corresponding estimates for humans [15], [30], [32], which may reflect the substantially higher *N_e_* in wild house mice. By multiplying estimates of *ω_a_* by the number of sites in each category, we can calculate the numbers of adaptive substitutions in protein-coding genes, UTRs and CNEs. Only ∼20% of adaptive substitutions are inferred to occur in coding sequences, and the vast majority of the remaining 80% occur in noncoding DNA, mostly CNEs. This is largely driven by the far higher number of sites in CNEs than in protein-coding genes, since estimates of *ω_a_* are quite similar among the site categories. Estimates of the number of adaptive noncoding substitutions are likely to be underestimates if there are elements that experience high rates of adaptive evolution, or elements specific to the murid lineage that we failed to identify on the basis of conservation. Additionally, there are many mammalian elements identified as part of the ENCODE project [6] lacking conservation that show reduced diversity in humans [44]. This has been interpreted as a signature of weak purifying selection [44], but a more plausible explanation is that the effect is attributable to selection on linked sites (see below).

### Nucleotide diversity around amino acid substitutions

Sattath et al. [35] have demonstrated the existence of reductions in nucleotide diversity in *D. simulans* close to sites that show an amino acid substitution between *D. melanogaster* and *D. simulans*. In contrast, synonymous substitutions do not show diversity reductions, implying that recent selective sweeps at nonsynonymous sites have purged diversity at linked sites. A parallel study in humans by Hernandez et al. [36] observed reductions in nucleotide diversity of similar magnitudes close to synonymous and nonsynonymous substitutions between human and chimpanzee. In our study in wild mice, we see a similar pattern to that observed in humans. Furthermore, we have also shown that reductions in diversity of a similar magnitude can be observed close to non-substituted sites. These observations suggest that diversity reductions around recently substituted non-synonymous sites in mammals are principally caused by selective sweeps or BGS at closely linked functional sites rather than at the focal sites themselves. The contrast between *Drosophila* and mammals is likely to be explained by the 10-fold narrower scales over which diversity is purged close to amino acid substitutions in *D. simulans*, compared to humans and mice [35], [36], resulting in little power to apply the Sattath et al./Hernandez et al. approach in hominids or murids. This is corroborated by our observation that diversity reductions surrounding exons and CNEs are caused by the influence of selection on multiple, linked elements and that little of the reductions can be explained by selection within the focal elements themselves. Hernandez et al. [36] argue that their observations suggest a lack of complete selective sweeps in humans. Although this may be true, the conclusion does not necessarily follow from their analysis.

### Background selection

To investigate whether the BGS model on its own can explain the drops in diversity around exons and CNEs, we obtained predictions of relative diversity in the genome using the approach and software described by McVicker et. al. [39] to find the parameters of a distribution of selection coefficients for nonsynonymous and CNE sites that best fit the genome-wide distribution of nucleotide diversity. We found that the best-fitting model accurately predicts the depth of the reductions in diversity in the immediate flanks of exons and CNEs, but under-predicts the width of reductions observed in the flanks of exons (Figure 4). An explanation for our failure to accurately predict the width of reductions in *π*/*d* in the flanks of exons is that recurrent selective sweeps may play a role in explaining the observed patterns of diversity around exons.

We investigated whether the inferred DFE from the BGS model is consistent with the amount of diversity observed within exons and CNEs themselves. We calculated the diversity predicted at nonsynonymous sites and CNEs, from the DFEs obtained from the best fitting BGS model of McVicker et al. [39]. Assuming that alleles act additively, the estimated DFEs predict a nucleotide diversity of 0.32 and 0.45 within exons and CNEs, respectively. The BGS model therefore overpredicts diversity at nonsynonymous sites (the observed values are 0.14 for 0-fold sites and 0.18 for 2-fold sites), but is close to the observed value of 0.42 for CNEs. We then used ML estimates of the DFE obtained from DFE-alpha to predict drops in diversity around exons and CNEs under a BGS model (Material and Methods). The resulting *r^2^* of the model is lower than the best fitting model, but not substantially so (1.57 compared to 1.88 for 1 Kb windows and 0.35 compared to 0.44 for 200 bp windows). Furthermore, when binning data by distance from the nearest exon or CNE and averaging *π*/*d* within bins, the predicted mean *π*/*d* is almost indistinguishable from that obtained from the best fitting model (Figure S4). Thus, we conclude that diversity data from within selected elements is largely consistent with deleterious mutations causing the observed reductions in *π*/*d* observed in the flanks of CNEs, but is inconsistent with reductions observed in the flanks of exons. This analysis can only qualitatively compare the fit of different models, and it is not possible to exhaustively explore all models. However, we have found that two models to predict diversity in the flanks of exons and CNEs that use very different information (the best fitting BGS model and a BGS model parameterised using DFEs inferred from DFE-alpha) give similar predictions, and both provide a poor fit to the pattern of diversity reduction around exons.

### Quantifying adaptive coding and noncoding fitness change

We have obtained evidence that there are many more adaptive noncoding than coding nucleotide substitutions. This does not necessarily imply, however, that noncoding substitutions dominate adaptive change, because the fitness effects of coding substitutions could be larger than those of noncoding mutations. Patterns of diversity around sites under positive selection shed some light on this issue, but for two reasons, it is not possible to reach a firm conclusion.

First, the rate of adaptive fitness change (*ΔW*) depends on the distribution of fitness effects of advantageous mutations (*f*(*s_a_*)). Consider a model in which adaptive mutations occur at a rate *μ_a_* at *n_a_* sites and are fixed with probability *u*(*s_a_*):
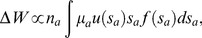
(2)Assuming that selection is strong relative to genetic drift, equation (2) becomes
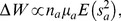
(3)which is proportional to the additive genetic variance for fitness from new advantageous mutations. Prediction of *ΔW* therefore requires knowledge of the average squared effect of an advantageous mutation, but we have not attempted to estimate this.

Second, adaptive evolution may be driven by complete selective sweeps originating from recent, novel mutations, from partial sweeps involving older, standing genetic variation or from some combination of these. The pattern of diversity around sites that have experienced positive selection is influenced by whether sweeps have been full or partial [45]. Recent evidence suggests that partial sweeps comprise a substantial proportion of adaptive events in humans [46].

If we assume that adaptation is dominated by complete sweeps, our results would imply that positive selection is substantially stronger on coding than on CNE mutations. This is firstly because BGS fails to account for the wide mean diversity reductions around exons (unlike CNEs), and secondly because the width of a region purged of variation from new mutations by positive selection is predicted to be proportional to *s_a_* [37]. Diversity is reduced by 50% around single exonic and CNE sites at distances of ∼38 Kb and 3 Kb, respectively, implying that the strength of selection on advantageous mutations in exons could be substantially higher than in CNEs. Our results therefore lend some support to the idea that, although there are many more adaptive noncoding changes, the net effect of coding change may exceed that of noncoding fitness change. However, it is not possible to exclude the existence of infrequent substitutions dominating adaptive fitness change involving alleles with large fitness effects that do not show up in the average patterns of diversity drops around selected sites.

## Materials and Methods

### Wild house mouse samples

Of 38 *M. m. castaneus* individuals from Himachal Pradesh, India [18], 21 were identified as belonging to a single cluster, labelled “North-West”, based on a STRUCTURE analysis [47] of 60 microsatellite loci. Protein-coding gene sequences of 15 of these individuals [2] suggested that one individual (H10) showed signs of inbreeding, having a substantially higher proportion of homozygous SNPs than other individuals, and was excluded from further analysis. We selected ten individuals for whole genome sequencing (individuals H12, H14, H15, H24, H26, H27, H28, H30, H34 and H36). We also sequenced a single individual of *Mus famulus*, originating from Tamil Nadu, India (locality Kotagiri), obtained from the Montpellier Wild Mice Genetic Repository, as an alternative outgroup to the rat.

### Genome sequencing and alignment

For each individual, five standard Illumina paired-end sequencing libraries were made with fragment sizes from 300–550 bp. We generated between 21–42× mapped sequence coverage (average 29×) across the samples (Table S9). The libraries were run at a mixture of 76, 100 and 108 bp read lengths on the Illumina GAIIx and HiSeq platforms. In order to check the identity of all of the Illumina sequencing lanes, we used a set of SNPs previously identified by Sanger sequencing of the same individuals [2]. This was done by using SAMtools mpileup (v0.1.16) in conjunction with BCFtools and GLFtools [48] to generate genotype likelihoods for each sample. All lanes were confirmed to be the correct genotype.

The *M. m. castaneus* Illumina sequencing reads were aligned to NCBIM37/mm9 unmasked reference genome with SMALT (http://www.sanger.ac.uk/resources/software/smalt/) using the following parameters: -k 13 -s 6. The individual lane BAM files were merged to the library level, PCR duplicates were marked using Picard (http://picard.sourceforge.net/), and a single merged BAM file was produced per sample. All of the sequencing data are available from the European Nucleotide Archive via accession ERP000231.

### SNP calling

We created bcf (genotype likelihood) files for each chromosome from the individual BAM files using ‘samtools mpileup’ with options -D -S -g -m 2 -F 0.0005 -P ILLUMINA [48]. We then used ‘bcftools view’ with options -A -g to obtain SNP calls for every site in the genome. bcftools allows the specification of a prior site frequency spectrum (SFS), which can improve genotype calls at each site. We obtained an approximate prior SFS for the genome using an iterative approach (see http://samtools.sourceforge.net/mpileup.shtml). We used bcftools to estimate a posterior SFS for all sites on chromosome 1, then used the SFS as a prior (using option -P) for a second call to bcftools, and iterated until the prior and posterior converged. The final posterior SFS was then used as a prior to obtain genotype calls for the whole genome, which were used to obtain site frequency spectra for specific genomic regions. We called all genotypes using an approximate *M. m. castaneus* reference sequence, which is identical to the NCBIM37/mm9 reference sequence, but with all SNPs at a frequency of >0.5 replaced with the major allele observed amongst the *M. m. castaneus* individuals. This reduced the number of SNP calls representing fixed differences between the mouse reference and the *M. m. castaneus* sequences and reduced the number of triallelic SNP calls (which can arise if a variant in *M. m. castaneus* also has a fixed difference to the reference). We filtered genotype calls having no mapped reads or where the Hardy-Weinberg equilibrium P-value reported by SAMtools (from a χ^2^ based test) was less than 0.0002.

In principle, our approach for inferring the SFS (by iterating the prior and posterior SFSs) should be close to optimal, since it makes most efficient use of the information on each individual's genotype. However, we obtain very similar inferred SFSs for the genome as a whole if we infer the allele frequency at each site by maximum likelihood using SAMtools [48] or by simply using the genotype calls for each individual (Figure S5A). We also investigated the effect on the inferred SFSs of removing sites with low coverage (measured as total coverage across all individuals) (Figure S5B). As expected, given that mean coverage was ∼30× and more than 80% of sites had coverage >10× in all individuals (Table S9), there was very little effect of removing sites with low coverage (up to a total coverage of 100 reads across all individuals). We observed a skew towards low frequency variants as the severity of filtering of the data increased. However it is possible that the sites that pass these stringent filters represent a biased set of sites in terms of diversity and allele frequency, as argued previously [49].

### Construction of the *M. famulus* genome sequence

*M. famulus* is divergent from *M. m. castaneus* and the *M. m. musculus* reference sequence and assembly of its genome sequence is therefore worth some special consideration. Specifically, divergent regions in the genome will reduce the efficacy or accuracy of the final sequence because reads with too many differences to the reference cannot be mapped properly to the reference genome. To mitigate this effect, we used an ‘iterative mapping’ approach where successive rounds of read mapping are conducted and after each iteration a new genome sequence is generated for use in the next iteration. In effect, we are converting the original reference genome to a *M. famulus* reference genome by changing the divergent sites to match those from *M. famulus*. Therefore, regions of high divergence where reads cannot be aligned initially may eventually be assembled as divergent sites are eliminated from the reference.

We aligned each of the lanes of data to the reference genome independently using BWA v0.5.9 [50]. We used SAMtools v0.1.16 mpileup to call variant SNPs and converted variant positions in the reference to match the all high quality homozygous variant calls (genotype quality, GQ>40). With this approach we ignored all shared polymorphisms that are heterozygous in our *M. famulus* sample and more importantly we also ignore potential indel divergence. We discarded indels and SNPs neighbouring indels to avoid converting regions of the genome where read mapping has erroneously generated indels due to repeats and to retain the same position indices of genomic features as the reference genome. The new reference was then used to repeat this process.

The most improvement in terms of positions covered and reads mapped occurred in the first and second iteration, after which the gains made with successive iterations plateaued. We carried out a total of five iterations over which the number of reads mapped increased from 72.6% to 84.0% and the median coverage improved from improved from 23× to 25×. After five iterations we called the final genotype of the *M. famulus* genome using the same methods described for the *M. m. castaneus*.

### Protein-coding genes and UTRs

We obtained gene coordinates from the Ensembl database version 62 for a total of 18,761 autosomal protein-coding genes, which are orthologous between mouse and rat. We used these to obtain the exonic sequence of the canonical spliceform of each gene for mouse and rat, and constructed sequences for *M. famulus* and *M. m. castaneus* based on the genotype calls. In order to preserve the reading frame, we constructed alignments using the translated amino-acid sequences and back-translated to the DNA sequence. We considered two classes of nonsynonymous sites: zero-fold and two-fold degenerate (for two-fold sites, only transversions were considered as nonsynonymous). However, in order to be able to construct an SFS for two-fold nonsynonymous we require an appropriate estimate of the number of invariant sites. To do this, we calculate the approximate fraction of two-fold sites at which a mutation would be nonsynonymous. We assume all transversions are nonsynonymous and calculate the ratio of transitional and transversional mutations from that observed at four-fold degenerate sites across all genes in a comparison of *M. m. castaneus* and rat. UTRs were identified as sequences annotated as being transcribed, but not forming part of a coding sequence.

### CNEs

We identified conserved noncoding elements (CNEs) in the mouse genome using phastCons [21]. We ran phastCons on multiz alignments of 28 vertebrate genomes based on Build 36.1/hg18 version of the human genome (see http://hgdownload.cse.ucsc.edu/goldenPath/hg18/multiz28way). We restricted the phastCons analysis to the data from placental mammals only and also excluded information from the mouse/rat lineage, thereby avoiding an ascertainment bias which could affect divergence within the mouse/rat lineage. We used parameters for phastCons that have been tuned to produce ∼5% conserved elements in the genome (expected-length = 45, target-coverage = 0.3, rho = 0.31) and that were used to produce the conservation scores and “most conserved” tract for the UCSC genome browser. Having identified CNEs in the human genome, we mapped the human coordinates onto the NCBIM37/mm9 mouse reference genome using the UCSC liftOver tool.

We subdivided CNEs into those proximal to exons (within 20 Kb of an exon, pCNEs) and those distal to exons (dCNEs, more than 20 Kb away from any exon). A 20 Kb cutoff was chosen such that dCNEs would be located outside of the flanks of exons that show reduced levels of *π*/*d* (see Figure 1). As such, dCNEs can be considered to be outside the shadow of protein-coding exons, allowing us to examine patterns of nucleotide diversity within CNEs and their flanks independently from protein-coding exons and their flanks.

### Neutral reference for CNEs

We attempted to obtain an appropriate neutral reference sequence to use when inferring rates of adaptation from our analysis of the SFS for CNEs. Ideally, this neutral reference sequence should be free from direct selection and tightly linked to the focal sequence such that the neutral reference and focal sequence can be assumed to share the same genealogy. We used sections of the genome near to CNEs, but far enough away such that mean divergence to rat approximates that for ancestral repeats (0.168 at non-CpG-prone sites) which occurs at ∼500 bp away from CNEs on average (Figure 1), Assuming that ancestral repeats are a good *a priori* candidate for neutrally evolving sites, as has been previously suggested [26], then this would imply that these regions are, on average, evolving neutrally. For each CNE we selected two sections split equally between a section upstream of the CNE and a section downstream of the CNE, each offset from the CNE start/end by 500 bp, such that the total length of the two sections was equal to the length of the CNE. From these sections of sequence, we masked any other CNEs and any annotated exons.

### Identification of ancestral repeat sequences

Remnants of transposable element repeat sequences that were inserted prior to the split of mouse and rat (ancestral repeats) provide a good choice for putatively neutral sequence, as mutations within these sequences are unlikely to affect host fitness. In order to identify potential ancestral repeat sequences, we obtained a set of coordinates of all identified repeat sequences in the mouse genome from Ensembl and removed from this set any repeat sequences that failed to align well with rat (those whose alignments contained >50% gaps or whose mean divergence, after applying a Kimura 1980 [51] correction for multiple hits, exceeded 1).

### Inference of the frequency of adaptive substitutions using the folded site frequency spectrum (SFS)

Folded SFSs for a class of selected sites (nonsynonymous sites, UTRs or CNEs) and a putatively neutral reference class were analysed by DFE-alpha [28], [32]. Our linked neutral reference class of sites, which were four-fold degenerate sites or intronic ARs for protein-coding genes and UTRs and sequences flanking CNEs (masked for any annotated exons or other CNEs) for CNEs. DFE-alpha uses maximum likelihood to fit a demographic model to the neutral site data, involving a step change in population size. Assuming the estimated parameters for the demographic model (Table S1), a gamma distribution of fitness effects (DFE) of new deleterious mutations is fitted to the selected site class. We assume that strongly advantageous mutations are sufficiently rare as to make a negligible contribution to polymorphism. In this analysis, it is not possible to infer the frequency of slightly advantageous mutations, since their contribution to the estimated DFE is effectively indistinguishable from that of slightly deleterious mutations [43]. Their contribution to estimates of α are therefore disregarded. From the estimated DFE parameters, the mean fixation probability, *u_n_*, of a deleterious mutation relative to a neutral mutation is calculated (Table 1). The proportion of adaptive substitutions is α = (*d_n_*−*d_s_u_n_*)/*d_n_*, and the relative rate of adaptive substitution is *ω_a_* = (*d_n_*−*d_s_u_n_*)/*d_s_*, where *d_n_* and *d_s_* are nucleotide divergences for the selected and neutral site classes, respectively, corrected for the contribution of polymorphism under the assumption of equal nucleotide diversity in the focal species and the outgroup [52]. Estimates for nonsynonymous sites are generally slightly lower than a previous study based on a small set of genes [2], but confidence limits overlap when using the same outgroup species (*M. famulus*) (Table S2).

### Nucleotide diversity around *M. m. castaneus* - *M. famulus* nonsynonymous substitutions

Attempts have previously been made to infer the strength of positive selection by comparing reductions in *π/d* around selected and putatively neutral sites that have experienced a substitution between related species [35], [36]. The principle of this approach is that if a large fraction of substitutions at selected sites have been driven to fixation by recent positive selection from new variation, then there will be a signature of reduced diversity around the locations of these substitutions, but this reduction is not expected to be observed (or to be as strong) in the regions surrounding neutral substitutions. Quantifying reductions in *π/d* around nonsynonyomous and synonymous substitutions allows this hypothesis to be tested if synonymous substitutions are assumed to be neutral. Synonymous substitutions also provide a suitable control, since they are interdigitated with nonsynonymous sites.

To estimate reductions in *π/d* in the flanks of nonsynonymous and synonymous substitutions, we firstly identified the locations of all substitutions within coding sequences between *M. m. castaneus* and *M. famulus* (excluding any that were segregating in either species). Coding substitutions were identified using the Ensembl genome annotation while only considering canonical spliceforms. From the annotation, we identified substitutions at four-fold degenerate sites (which were considered synonymous) and those at zero-fold degenerate sites (considered nonsynonymous). Then, for 1 Kb non-overlapping windows up to 100 Kb away from each substituted site, we obtained estimates of neutral *π*, divergence from rat (*d*) and *π/d* (by excluding any sequence located within an exon or a CNE). Estimates of divergence between mouse and rat were obtained using the chained and netted whole genome alignments of mouse and rat (using rat genome version rn4). We averaged estimates of each statistic over windows at equivalent distances from each zero-fold or four-fold substituted site, and plotted these averages as a function of distance. For comparison, we also calculated *π/d* around non-substituted sites.

### Examining patterns of neutral nucleotide diversity around exons and CNEs

In order to investigate the influence of selection acting within exons and CNEs on patterns of nucleotide diversity (*π*) in other parts of the genome, we first divided the genome into non-overlapping windows of 200 bp or 1 Kb. For each window, we excluded any sites belonging to exons (protein-coding or UTR) or CNEs and then calculated estimates of *π*, divergence from the rat (*d*), and *π/d*. As above, divergence was obtained using the chained and netted whole genome alignments of mouse and rat. Windows for which *π* or *d* could not be calculated due to missing data (*e.g.* those lacking divergence data due to deletions in rat, insufficient coverage in *M. m. castaneus* or because they completely overlapped a CNE or exon) were excluded from further analysis. In total 72% of windows of length 200 bp and 80% windows of length 1 Kb were retained for further analysis.

### Estimating genetic distance

For each non-overlapping window, we calculated a genetic position on each chromosome (in cM) based on a genetic map [53]. Note that for ease of fitting of the models described below, we also scaled these genetic distances by multiplying by a constant factor of 1,708,728 (the average number of bp/cM in the genome over chromosomes 1 to 19, based on the genetic map) such that their magnitude was comparable to a physical scale in bp. We also obtained positions on the same scale for the start of end of each exon and CNE.

### Modelling *π/d* around the genome

To investigate patterns of *π/d* around exons and CNEs we attempted to model *π/d* calculated for non-exonic and non-CNE sites within 200 bp or 1 Kb non-overlapping windows throughout the genome (described above) as functions of distance to exons and CNEs. To do this we fitted a variety of models described below. In all cases we only considered data for positions within the neighbourhood of exons and CNEs (positions within 100 Kb of an exon and 10 Kb of a CNE, a selection that includes 64% and 63% of the useable non-overlapping windows described above).

Firstly, we fitted a number of simple linear models (using lm in the R statistical package) where *π/d* is modelled as a linear function of distance or log-distance to the nearest exon and nearest CNE (either on a physical or genetic scale). We tested whether adding length of the nearest exon and CNE as predictors substantially improved the fit of this model, since it might be expected that the observed reductions in diversity depend on the number of nearby selected sites. Secondly, we investigated a more biologically informative range of models where reductions in diversity decrease exponentially with distance from the nearest exon and CNE, following [36]. These non-linear models were fitted using nls in the R statistical package. We fitted functions of the form *π/d*∼*p_1_*(1−*p_2_*.exp(-*x*/*p_3_*)), where *x* is the (physical or genetic) distance from an exon or CNE and *p_1_*, *p_2_* and *p_3_* are estimated parameters. Under this model, *p_1_* can be interpreted as an estimate of the neutral or unreduced value of *π/d* as *x* tends to infinity, *p_2_* as the reduction in *π/d* when x = 0 and *p_3_* as quantifying the distance over which neutral *π/d* is recovered.

We also investigated two more models that allow us to take into account the individual effects of all partially-linked selected exonic and CNE sites on *π/d* for a given genomic window. In our first model, rather than assuming that diversity around selected *elements* decreases exponentially, we assume that diversity around all selected *sites* within selected elements decreases exponentially, and that effects from multiple selected sites on a given neutrally evolving genomic position combine multiplicatively. Therefore, *π/d* for a single neutral site can be written as a product over all linked selected sites:
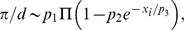
where *p_1_* is the “neutral” or unreduced value of *π/d*, *p_2_* is the reduction in diversity observed at a single selected site and *p_3_* measures the rate of decay. Assuming that individual reductions in *π/d* are small, then this can be approximated as:
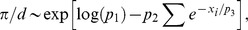
or for two classes of mutational effects (i.e., reductions due to exonic and CNE sites):
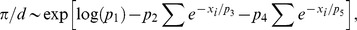
where there *n* and *m* are numbers of exonic and CNE sites respectively. We fitted this model to the data by least squares, by minimising the sum of squared deviations between the observed and predicted estimates of *π/d* for each non-overlapping window. For computational efficiency, we used predictions calculated at the midpoint of each non-overlapping window as a proxy for the average over the window.

### Background selection model

In our second model we attempted to predict *π/d* for each window by considering the reduction expected as a result of BGS, a process whereby variation is purged as a result of negative selection on linked loci [41]. We obtained estimates of the reductions in neutral diversity expected for exons and CNEs at the midpoint of each of our genomic windows using the approach and software described in ref. 39. For this model we assume a constant mutation rate, which we estimated by dividing a Jukes-Cantor-corrected estimate of divergence between mouse and rat at four-fold degenerate sites of 0.182 by an estimate of the number of generations separating mouse and rat. We assume that mouse and rat diverged either 12 MYA and have on average two generations per year, giving a separation of 48 generations and a mutation rate estimate of 3.79×10^−9^. We account for the fact that only a fraction of changes at exonic sites are nonsynonymous by multiplying the mutation rate for exonic regions by the average fraction of nonsynonymous sites per exon (0.718). We assume a recombination rate of 0.528 cM/Mb [42].

Predictions for the reduction in neutral diversity were obtained separately for exons and CNEs and we assume that reductions from each element type combine multiplicatively to give the total reduction for a given genomic position. To compare the predicted reductions to our estimates of *π/d* we scaled the predicted reductions by multiplying by the unreduced value for *π/d* which we estimate by minimising the sum of squared differences between the scaled predicted values and the observed (equivalent to a linear regression of the the observed on the predictions with a zero intercept). The sum of squared deviations between the observed and scaled predicted reductions can be used to obtain an *r* ^2^ for the model. To obtain our best fitting model of BGS, we explored a range of exponential DFEs for both exons and CNEs (over several orders of magnitude) and found the parameters of these distributions that maximized the *r* ^2^ of the model. We also obtained predictions for the reduction in neutral diversity expected from the DFEs inferred for nonsynonymous sites and CNEs from DFE-alpha (for non-synonymous sites, we use parameters inferred for zero-fold degenerate sites). In this case, due to numerical instability it was not possible to obtain predictions from the BGS model using the ML *β* parameters obtained from DFE-alpha (i.e. 0.11 for zero-fold degenerate sites and 0.16 for CNEs). To circumvent this issue we obtained estimates for the mean effect of a deleterious mutation using DFE-alpha assuming a fixed *β* = 0.25 (*N_e_s* = 1.1×10^3^ for zero-fold sites and *N_e_s* = 16 for CNEs). Similar results were also obtained when using DFE-alpha estimates where we set *β* = 1.0, suggesting the predictions from the BGS model are not sensitive to the shape parameter assumed (data not shown).

### Ethics statement

All animal work followed the legal requirements, was registered under number K-13/14-05 (Evolutionary Genomics), and was approved by the animal ethics commission of the University of Cologne (Germany).

## Supporting Information

Figure S1 Observed and expected (i.e., fitted) folded SFSs for neutral (A) and selected (B) categories of sites under a two-epoch model.
(TIFF)

Figure S2 Cumulative frequency distribution-of between-CNE and between-exon lengths in the genome. When calculating the between exon length distribution, we only considered coding exons from canonical transcripts, to be consistent with other analyses.
(TIFF)

Figure S3 Number of 200 bp genomic windows as a function of distance from the middle of the window to the nearest exon and nearest CNE.
(TIFF)

Figure S4 Predicted and observed diversity in genomic windows binned by absolute distance from exon and CNE boundaries. Mean observed reductions are shown as black dots. Mean predicted values from a model assuming that reductions in diversity for each selected site are exponentially distributed (model C) are shown as blue lines. Mean predictions from a best fitting model of background selection (assuming an exponential distribution of effects) are shown as green lines (BGS best) and mean predictions from a background selection model using the DFEs inferred from DFE-alpha are shown as red lines (BGS DFEalpha).
(TIFF)

Figure S5 Inferred site frequency spectra for chromosome 1. A. SFS inferred under three different approaches: genotype calls (SFS inferred using genotype calls for each individual from SAMtools), SF1 (SFS inferred using allele frequency estimated by maximum likelihood using SAMtools and stored in the SF1 field of the INFO column in the VCF) and Bayesian (posterior SFS, inferred from SAMtools, using an iterated prior as described in the text. B. SFS inferred using genotype calls and applying a minimum total depth of coverage filter (i.e. summed coverage over all individuals).
(TIFF)

Table S1 Demographic parameter estimates.
(PDF)

Table S2 Estimates of selection parameters from a DFE-alpha analysis.
(PDF)

Table S3 Estimated DFE parameters and rates of adaptive molecular evolution obtained when using ancestral repeats located within introns as a neutral standard.
(PDF)

Table S4 Changes in log likelihood (Δlog*L*) between 2-epoch and 3-epoch demographic models and parameter estimates for 3-epoch model.
(PDF)

Table S5 Demographic and selection parameter estimates for pCNEs and dCNEs.
(PDF)

Table S6 Estimated parameters of a DFE model with three discrete mutation fitness effect bins.
(PDF)

Table S7 Estimated reductions in diversity in the flanks of exons and CNEs.
(PDF)

Table S8 Parameter estimates of models to of *π*/*d* calculated in 200 bp for 1000 bp non-overlapping windows around the genome.
(PDF)

Table S9 Coverage statistics for sequences of 10 *M. m. castaneus* and one *M. famulus* individual.
(PDF)

Text S1 Additional details of methodology.
(PDF)
