# Supplementary figures and images for: Contributions of Protein-Coding and Regulatory Change to Adaptive Molecular Evolution in Murid Rodents
Source: PLoS Genet. 2013 Dec 5;9(12):e1003995. doi: 10.1371/journal.pgen.1003995 (PMC3854965; doi:10.1371/journal.pgen.1003995)

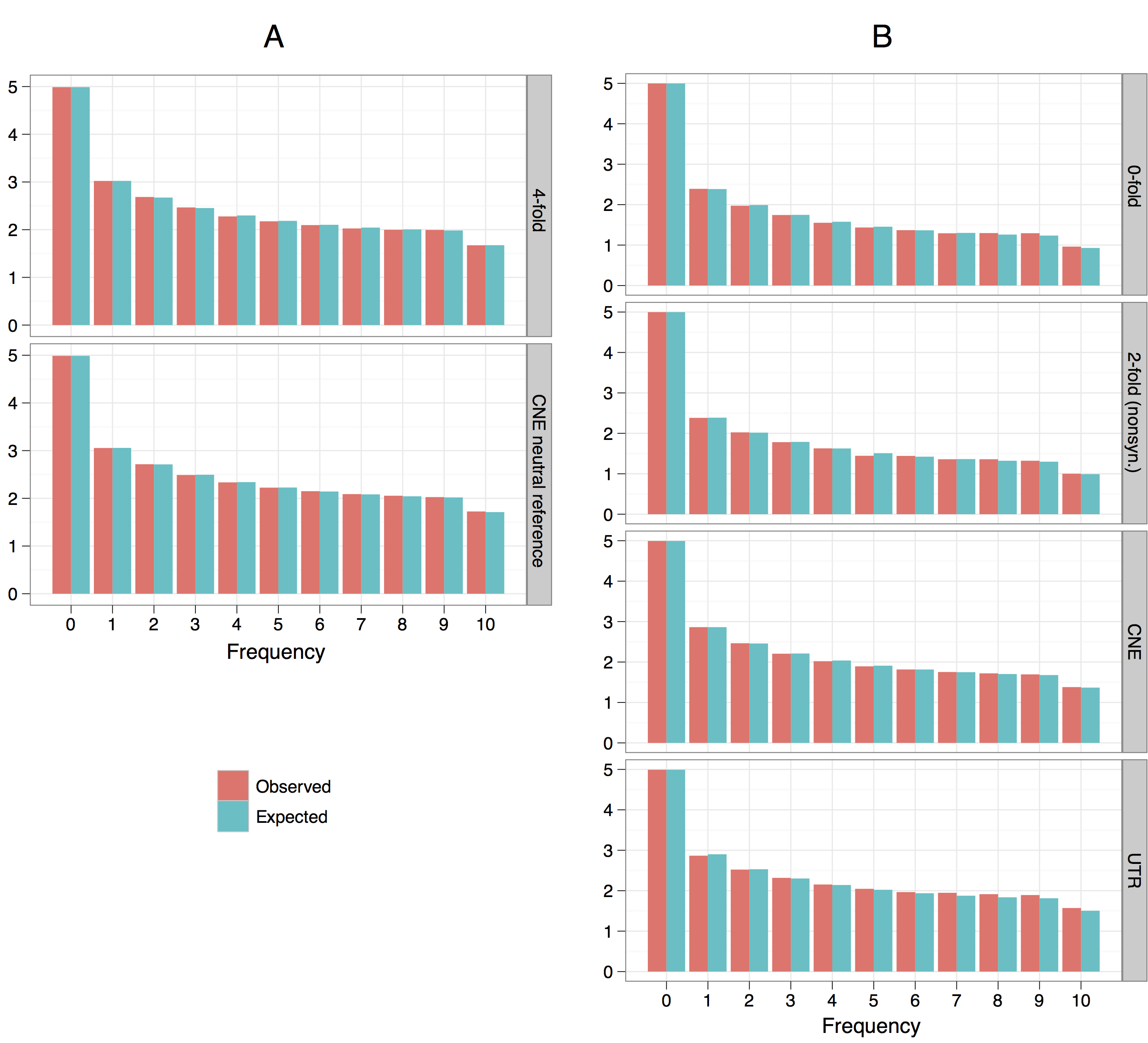

Supplement: Figure S1 — Observed and expected (i.e., fitted) folded SFSs for neutral (A) and selected (B) categories of sites under a two-epoch model. (TIFF) [file pgen.1003995.s001.tiff]

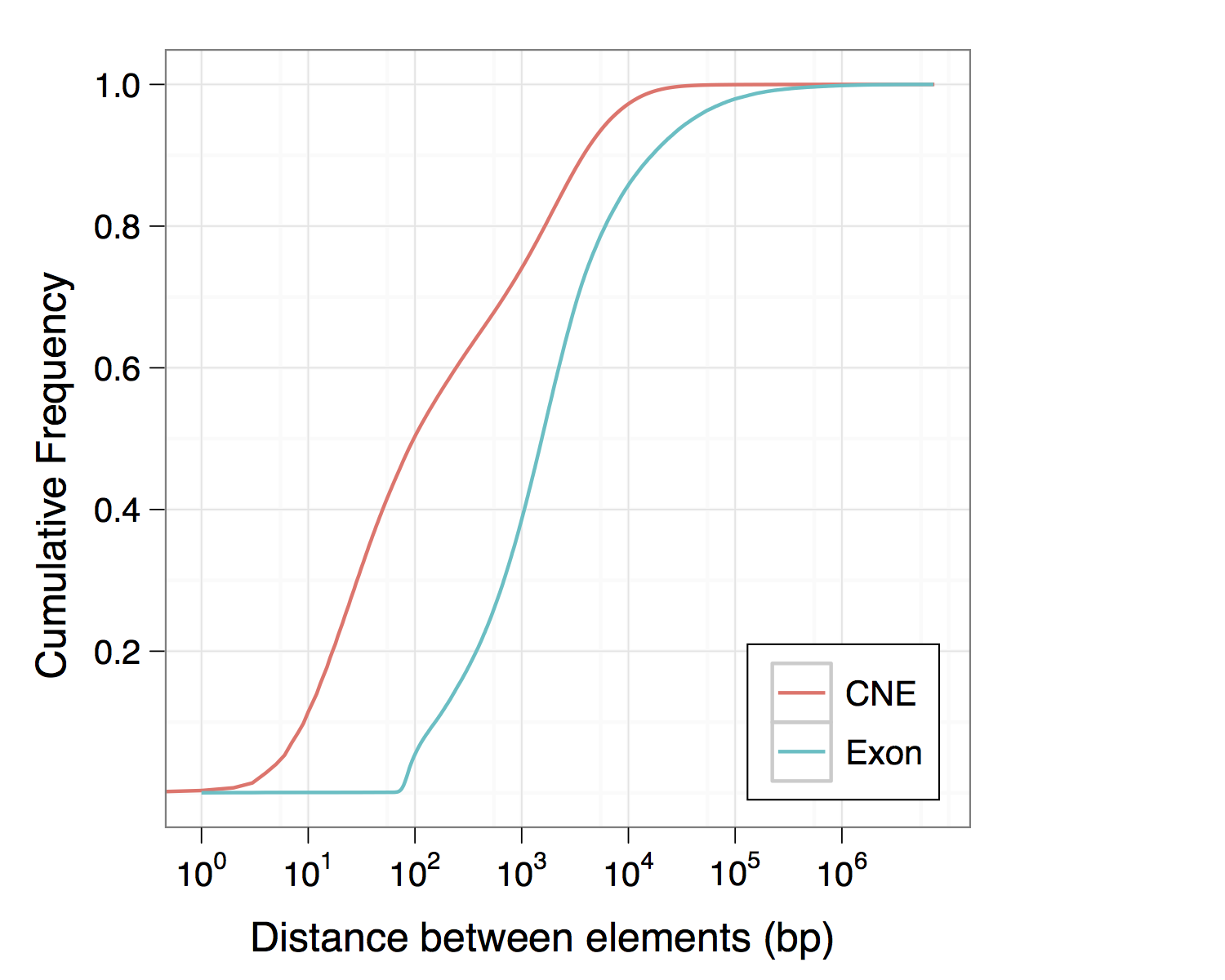

Supplement: Figure S2 — Cumulative frequency distribution-of between-CNE and between-exon lengths in the genome. When calculating the between exon length distribution, we only considered coding exons from canonical transcripts, to be consistent with other analyses. (TIFF) [file pgen.1003995.s002.tiff]

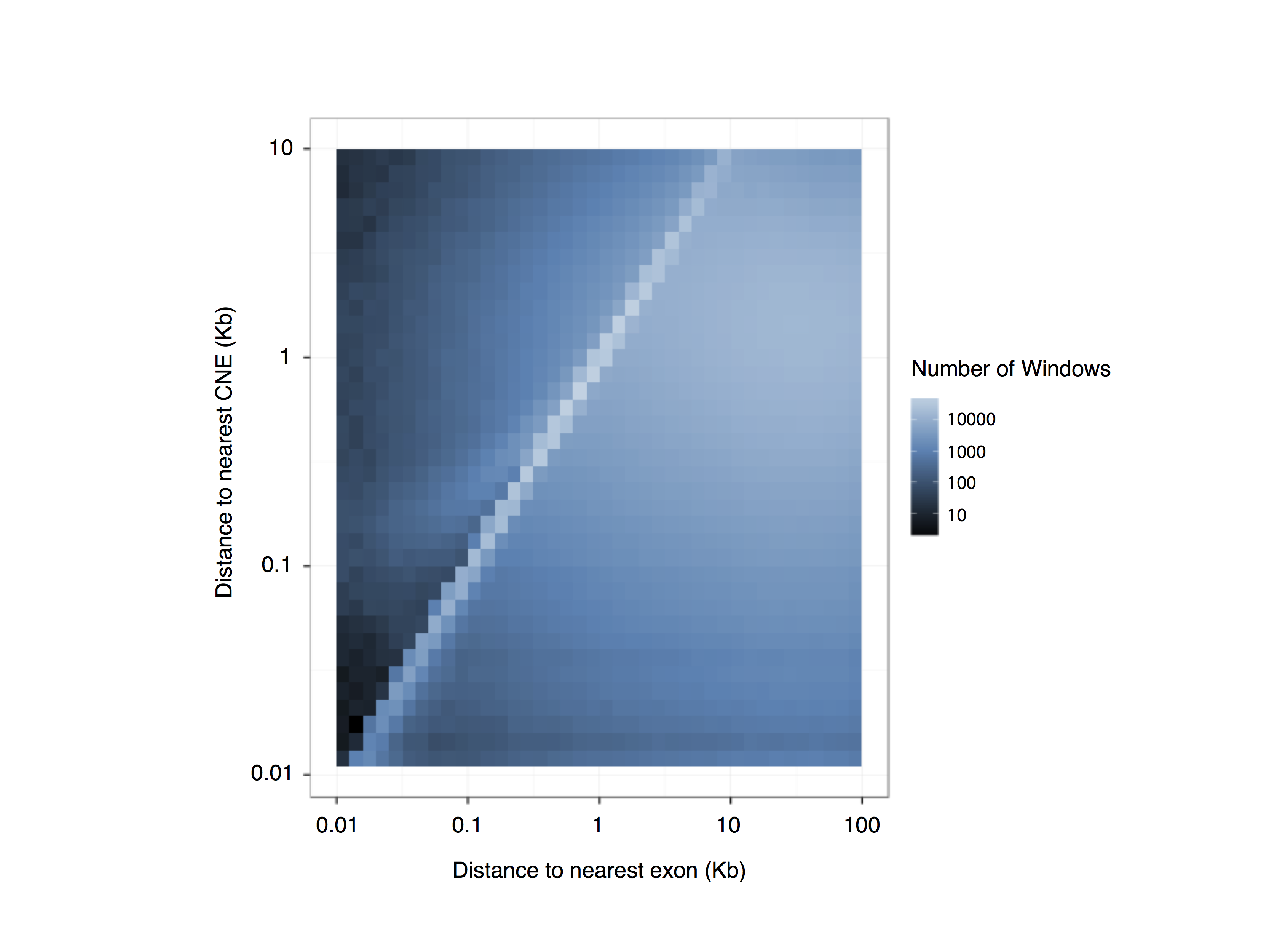

Supplement: Figure S3 — Number of 200 bp genomic windows as a function of distance from the middle of the window to the nearest exon and nearest CNE. (TIFF) [file pgen.1003995.s003.tiff]

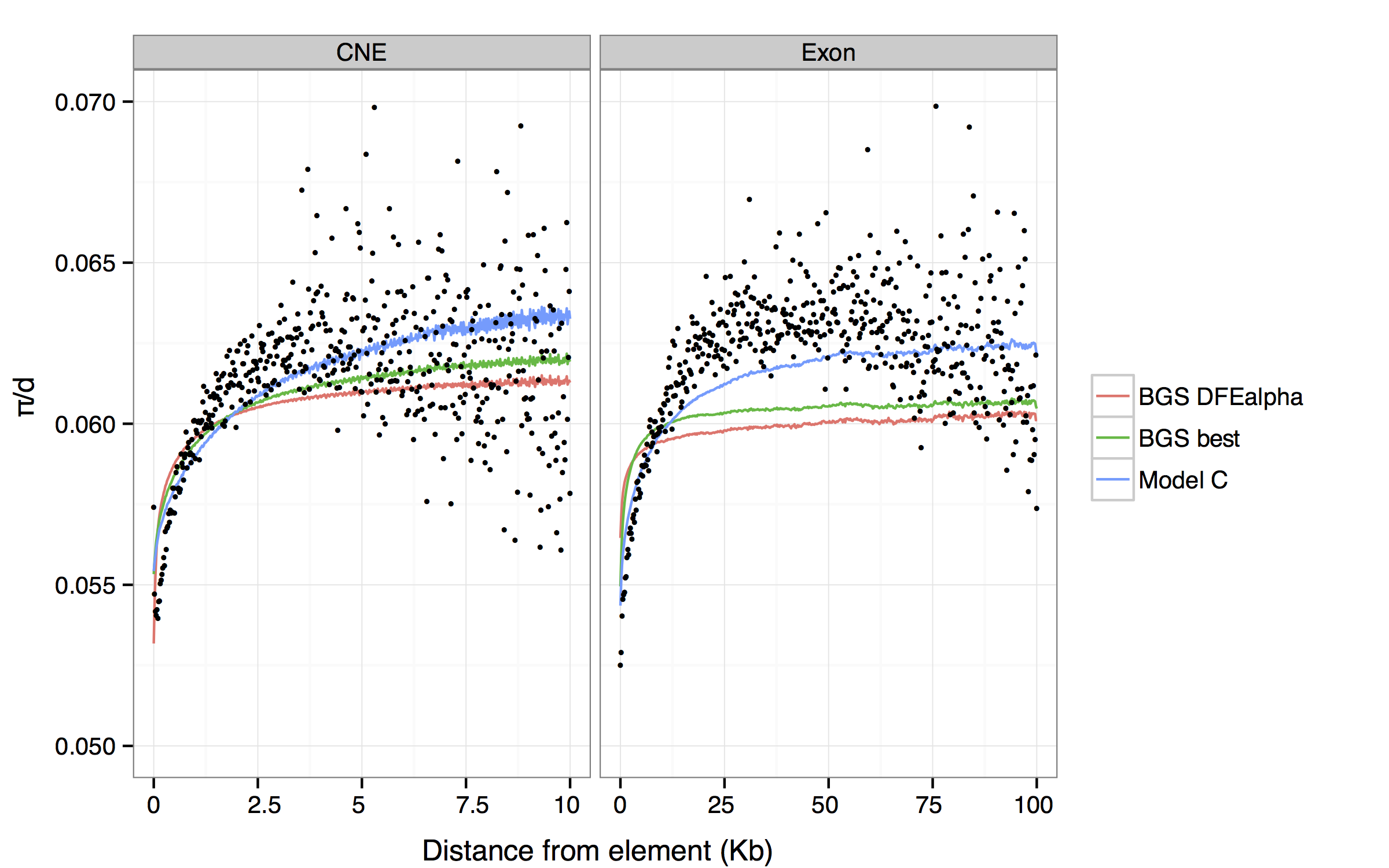

Supplement: Figure S4 — Predicted and observed diversity in genomic windows binned by absolute distance from exon and CNE boundaries. Mean observed reductions are shown as black dots. Mean predicted values from a model assuming that reductions in diversity for each selected site are exponentially distributed (model C) are shown as blue lines. Mean predictions from a best fitting model of background selection (assuming an exponential distribution of effects) are shown as green lines (BGS best) and mean predictions from a background selection model using the DFEs inferred from DFE-alpha are shown as red lines (BGS DFEalpha). (TIFF) [file pgen.1003995.s004.tiff]

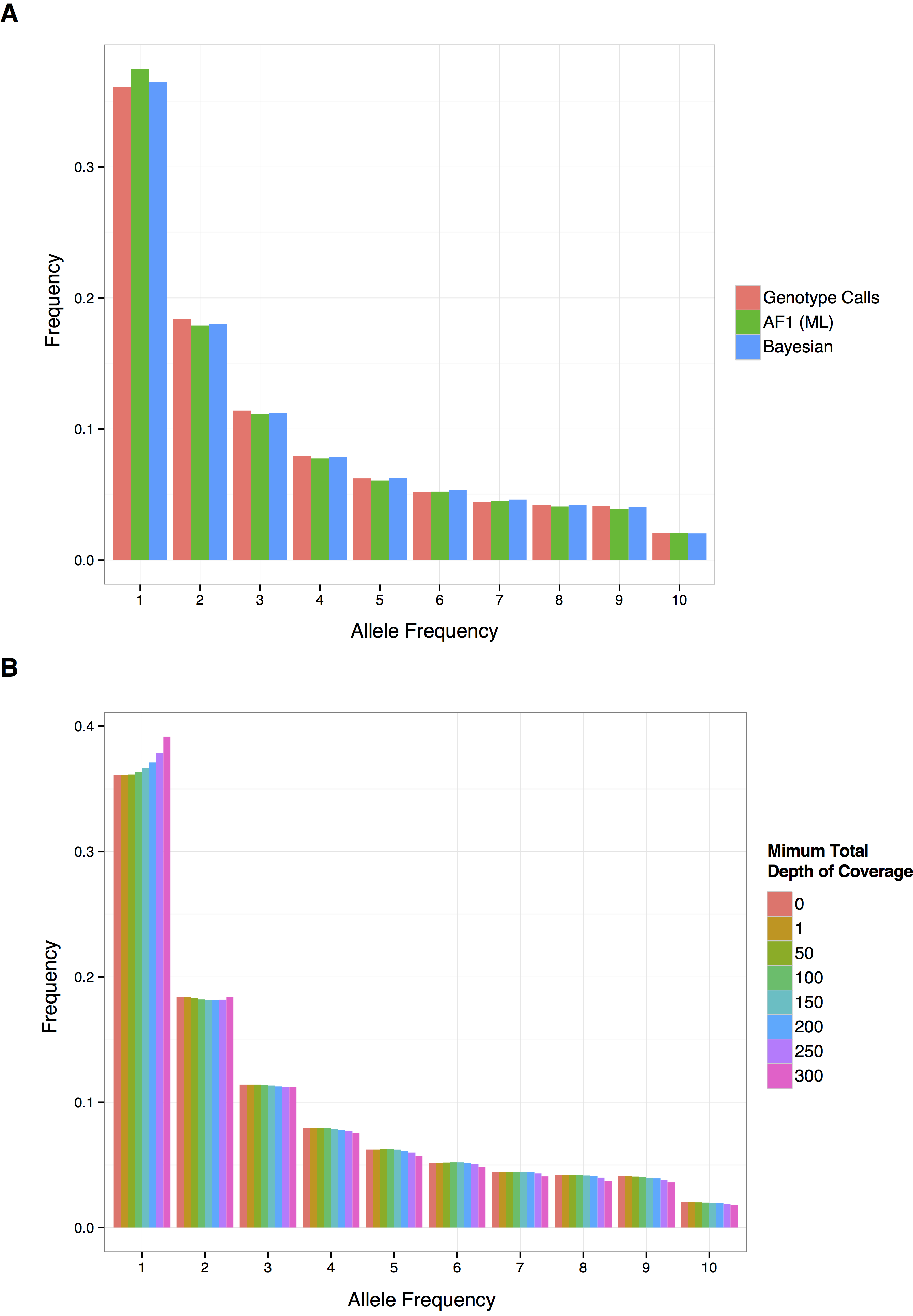

Supplement: Figure S5 — Inferred site frequency spectra for chromosome 1. A. SFS inferred under three different approaches: genotype calls (SFS inferred using genotype calls for each individual from SAMtools), SF1 (SFS inferred using allele frequency estimated by maximum likelihood using SAMtools and stored in the SF1 field of the INFO column in the VCF) and Bayesian (posterior SFS, inferred from SAMtools, using an iterated prior as described in the text. B. SFS inferred using genotype calls and applying a minimum total depth of coverage filter (i.e. summed coverage over all individuals). (TIFF) [file pgen.1003995.s005.tiff]
