# Supplementary material for: Contributions of Protein-Coding and Regulatory Change to Adaptive Molecular Evolution in Murid Rodents
Source: PLoS Genet. 2013 Dec 5;9(12):e1003995. doi: 10.1371/journal.pgen.1003995 (PMC3854965; doi:10.1371/journal.pgen.1003995)
Supplement: Table S1 — Demographic parameter estimates. (PDF) [file pgen.1003995.s006.pdf]

Table S1. Demographic parameter estimates for 2-epoch demographic model and differences in log likelihood between 2-epoch and constant population (*ΔlogL*) model fitted to folded SFSs for putatively neutral classes of sites when analysing non-CpG-prone sites.

Site Class	N_2/N_1	t_2/N_1	*ΔlogL*
Synonymous	2.79<br>[2.79,3.07]	1.47<br>[1.33,2.13]	1719
CNE neutral reference	2.79<br>[2.79,2.79]	1.61<br>[1.55,1.69]	32321
pCNE neutral reference	2.79<br>[2.79,2.79]	1.76<br>[1.63,1.88]	11798
dCNE neutral reference	2.79<br>[2.79,2.79]	1.55<br>[1.47,1.63]	20536
