# Supplementary material for: Contributions of Protein-Coding and Regulatory Change to Adaptive Molecular Evolution in Murid Rodents
Source: PLoS Genet. 2013 Dec 5;9(12):e1003995. doi: 10.1371/journal.pgen.1003995 (PMC3854965; doi:10.1371/journal.pgen.1003995)
Supplement: Table S2 — Estimates of selection parameters from a DFE-alpha analysis. (PDF) [file pgen.1003995.s007.pdf]

Table S2. Estimates of selection parameters from a DFE-alpha analysis of folded SFSs for non-CpG-prone sites for different site classes.

Site Class	$N_w s_{del}$	β	α (cas)	ω_a (cas)	α (fam)	ω_a (fam)	α (rat)	ω_a (rat)
Zero-fold	9.5e5 [3.5e7, 2.6e5]	0.11 [0.088, 0.13]	0.2 [0.16, 0.24]	0.042 [0.032, 0.049]	0.32 [0.29, 0.37]	0.075 [0.068, 0.095]	0.32 [0.28, 0.35]	0.077 [0.067, 0.087]
Two-fold (nonsyn.)	→∞ [→∞, 4.0e9]	→0 [→0, 0.066]	0.19 [0.15, 0.24]	0.046 [0.036, 0.059]	0.31 [0.29, 0.38]	0.089 [0.082, 0.12]	0.38 [0.36, 0.42]	0.12 [0.12, 0.14]
UTRs	250 [500, 140]	0.050 [0.050, 0.050]	0.039 [-0.0053, 0.083]	0.026 [-0.0035, 0.057]	0.21 [0.18, 0.28]	0.17 [0.15, 0.25]	0.19 [0.16, 0.23]	0.15 [0.12, 0.19]
CNEs	45 [50, 40]	0.16 [0.15, 0.17]	0.25 [0.23, 0.26]	0.13 [0.12, 0.14]	0.37 [0.36, 0.39]	0.24 [0.23, 0.25]	0.19 [0.18, 0.21]	0.097 [0.091, 0.10]
pCNE	81 [110, 63]	0.12 [0.11, 0.13]	0.23 [0.21, 0.25]	0.13 [0.12, 0.15]	0.35 [0.32, 0.37]	0.24 [0.22, 0.26]	0.18 [0.16, 0.19]	0.097 [0.086, 0.11]
dCNE	31 [34, 28]	0.19 [0.19, 0.20]	0.25 [0.24, 0.27]	0.12 [0.12, 0.13]	0.39 [0.37, 0.40]	0.23 [0.22, 0.24]	0.20 [0.19, 0.22]	0.094 [0.088, 0.10]

$N_w s_{del}$ is the scaled mean effect of a deleterious mutation. β is the shape parameter of gamma distribution. Estimates of α and ω_a are provided for three possible divergences: the *M. m. castaneus* only branch (using the inferred number of fixed differences from the unfolded SFS), *M. m. castaneus-M. famulus* (using mean divergence between *M. m. castaneus* and *M. famulus*) and *M. m. castaneus*-rat (using mean divergence between *M. m. castaneus* and rat). Note that in all cases, divergence is corrected for multiple hits using a Jukes-Cantor correction, and for *M. m. castaneus-M. famulus and M. m. castaneus*-rat we also correct divergence for the potential contribution of polymorphism [52] pCNEs and dCNEs are defined as CNEs located less than 20Kb and more than 20Kb from an annotated exon respectively. 95% bootstrap confidence intervals, shown in square brackets, were calculated by bootstrapping 1,000 times by gene (in the case of zero-fold and two-fold nonsynonymous sites and UTRs) or by 10,000bp sections of the genome (in the case of CNEs, pCNEs and dCNEs).
