# Supplementary material for: Contributions of Protein-Coding and Regulatory Change to Adaptive Molecular Evolution in Murid Rodents
Source: PLoS Genet. 2013 Dec 5;9(12):e1003995. doi: 10.1371/journal.pgen.1003995 (PMC3854965; doi:10.1371/journal.pgen.1003995)
Supplement: Table S3 — Estimated DFE parameters and rates of adaptive molecular evolution obtained when using ancestral repeats located within introns as a neutral standard. (PDF) [file pgen.1003995.s008.pdf]

Table S3. Estimated DFE parameters and rates of adaptive molecular evolution obtained when using ancestral repeats located within introns (excluding sites that may function as intronic splice sites, defined as the first and last 30bp of each intron) as a neutral standard.

Site Class	$N_w s_{del}$	β	α (cas)	ω_a (cas)	α (fam)	ω_a (fam)	α (rat)	ω_a (rat)
Zero-fold	3.1e4	0.17 [0.16, 0.18]	0.46 [0.44, 0.48]	0.11 [0.10, 0.11]	0.56 [0.53, 0.58]	0.16 [0.15, 0.16]	0.45 [0.42, 0.27]	0.10 [0.092, 0.11]
Two-fold (nonsyn.)	5.4e6 [9.3e8, 3.4e5]	0.11 [0.079, 0.13]	0.45 [0.41, 0.49]	0.12 [0.11, 0.4]	0.55 [0.51, 0.58]	0.18 [0.17, 0.20]	0.49 [0.46, 0.53]	0.15 [0.14, 0.16]
UTRs	5.4e3 [9.8e3, 3.1e3]	0.05 [0.05, 0.05]	0.27 [0.24, 0.30]	0.20 [0.18, 0.23]	0.40 [0.37, 0.42]	0.37 [0.33, 0.40]	0.25 [0.23, 0.28]	[0.19 [0.17, 0.21]
CNEs	25 [27,24]	0.23 [0.22, 0.24]	0.39 [0.38, 0.40]	0.21 [0.20, 0.22]	0.51 [0.50, 0.52]	0.35 [0.34, 0.36]	0.32 [0.31, 0.33]	0.15 [0.15, 0.16]
