# Supplementary material for: Contributions of Protein-Coding and Regulatory Change to Adaptive Molecular Evolution in Murid Rodents
Source: PLoS Genet. 2013 Dec 5;9(12):e1003995. doi: 10.1371/journal.pgen.1003995 (PMC3854965; doi:10.1371/journal.pgen.1003995)
Supplement: Table S4 — Changes in log likelihood (ΔlogL) between 2-epoch and 3-epoch demographic models and parameter estimates for 3-epoch model. (PDF) [file pgen.1003995.s009.pdf]

Table S4. Changes in log likelihood (ΔlogL) between 2-epoch and 3-epoch demographic models and parameter estimates for 3-epoch model.

**Site class**	**ΔlogL**	N_2	t_2	N_3	t_3
Four-fold	7.0	2	7.4	50	29
CNE neutral reference	45.1	40	542	110	52
