# Supplementary material for: Contributions of Protein-Coding and Regulatory Change to Adaptive Molecular Evolution in Murid Rodents
Source: PLoS Genet. 2013 Dec 5;9(12):e1003995. doi: 10.1371/journal.pgen.1003995 (PMC3854965; doi:10.1371/journal.pgen.1003995)
Supplement: Table S5 — Demographic and selection parameter estimates for pCNEs and dCNEs. (PDF) [file pgen.1003995.s010.pdf]

Table S5. Demographic and selection parameter estimates for pCNEs and dCNEs using putatively neutrally evolving standard sequences offset from the CNEs by 200, 500 and 1000bp. Estimates are obtained for non-CpG-prone sites only using rat as an outgroup.

Site Class	**Neutral reference offset (bp)**	N_2/N_1	t_2/N_1	$N_e s$	β	α **(rat)**	ω_a **(rat)**
dCNE	200	3.07	1.55	-39.9	0.153	0.150	0.0742
dCNE	500	2.79	1.55	-30.8	0.194	0.204	0.0937
dCNE	1000	2.79	1.77	-32.2	0.204	0.230	0.104
pCNE	200	2.79	1.33	-103	0.101	0.139	0.0796
pCNE	500	2.79	1.76	-81.4	0.120	0.177	0.0968
pCNE	1000	2.79	1.88	-77.6	0.126	0.184	0.0991
