# Supplementary material for: Contributions of Protein-Coding and Regulatory Change to Adaptive Molecular Evolution in Murid Rodents
Source: PLoS Genet. 2013 Dec 5;9(12):e1003995. doi: 10.1371/journal.pgen.1003995 (PMC3854965; doi:10.1371/journal.pgen.1003995)
Supplement: Table S6 — Estimated parameters of a DFE model with three discrete mutation fitness effect bins. (PDF) [file pgen.1003995.s011.pdf]

Table S6. Estimated parameters of a DFE model with three discrete mutation fitness effect bins (s=0, s=s_2 and s=1) with proportions (p_1, p_2 and p_3), the average fitness effect (weighted average of 0, s_2 and 1), the change in log likelihood from a gamma model of the DFE ($\Delta \log L = \log L_{discrete} - \log L_{gamma}$), the mean fixation probability (u_n) and estimates of α and ω_a. Estimates are obtained for non-CpG-prone sites only using rat as an outgroup.

Site Class	$N_e s_2$	p_1	p_2	p_3	***Mean*** $N_e s$	$\Delta \log L$	u_N	α (rat)	ω_a (rat)
Zero-fold	7.63	0.189	0.0276	0.786	143	18.7	0.187	0.224	0.053
Two-fold (nonsyn.)	27.7	0.209	0.0	0.791	144	-6.42	0.209	0.353	0.114
UTRs	2.47	0.72	0.0	0.282	51	-4.82	0.718	0.106	0.0851
CNEs	7.20	0.46	0.287	0.249	48.6	57.9	0.464	0.0677	0.0336
