# Supplementary material for: Contributions of Protein-Coding and Regulatory Change to Adaptive Molecular Evolution in Murid Rodents
Source: PLoS Genet. 2013 Dec 5;9(12):e1003995. doi: 10.1371/journal.pgen.1003995 (PMC3854965; doi:10.1371/journal.pgen.1003995)
Supplement: Table S7 — Estimated reductions in diversity in the flanks of exons and CNEs. (PDF) [file pgen.1003995.s012.pdf]

Table S7. Estimated reductions in diversity in the flanks of exons and CNEs. To quantify reductions in diversity we fitted the function $f(x)=A(1-B.\exp(-x/d))$ to mean π/d calculated for across non-overlapping windows flanking exons and CNEs separately by non-linear least squares. In all cases we excluded CNEs from the flanks of exons and any annotated exons from the flanks of CNEs. The categories are defined as follows:

1. CNEs: CNE flanks
2. CNEs (AR only): CNEs flanks restricted to ancestral repeats only
3. CNEs (exc. adjacent 500): CNEs flanks excluding the 500bp adjacent to each CNE excluded
4. CNEs (exc. adjacent 1,000): CNEs flanks excluding the 1,000bp adjacent to each CNE excluded
5. dCNEs: Flanking sequences of dCNEs only (CNEs located >20Kb from any exon)
6. mCNEs: Flanking sequences of mCNEs (see Supplementary Methods for description)
7. Exons: Exon flanks
8. Exons (AR only): Exon flanks restricted to ancestral repeats only
9. Exons (exc. CNE flanks): Exon flanks excluding not only CNEs, but also 500bp upstream and downstream of every CNE.

Site Class	***A***	***d***	***Width (d.ln2)***	***Depth (B)***
CNEs	0.0501	1.17	0.809	0.108
CNEs (AR only)	0.0507	1.19	0.827	0.0675
CNEs (exc. adjacent 500bp)	0.0501	1.19	0.825	0.107
CNEs (exc. Adjacent 1,000bp)	0.0501	1.24	0.859	0.103
dCNEs	0.0507	1.21	0.838	0.0671
mCNEs	0.0499	1.21	0.836	0.125
Exons	0.0511	13.0	9.04	0.152
Exons (AR only)	0.0530	12.5	8.65	0.156
Exons (exc. CNE flanks)	0.0513	10.6	7.35	0.150
