# Supplementary material for: Contributions of Protein-Coding and Regulatory Change to Adaptive Molecular Evolution in Murid Rodents
Source: PLoS Genet. 2013 Dec 5;9(12):e1003995. doi: 10.1371/journal.pgen.1003995 (PMC3854965; doi:10.1371/journal.pgen.1003995)
Supplement: Table S8 — Parameter estimates of models to of π/d calculated in 200 bp for 1000 bp non-overlapping windows around the genome. (PDF) [file pgen.1003995.s013.pdf]

Table S8. Parameter estimates of models to of π/d calculated in 200bp for 1000bp non-overlapping windows around the genome. The models are:

A: $\pi/d \sim p_1 + p_2 \log(d_{exon}) + p_3 \log(d_{CNE})$

B: $\pi/d \sim p_1(1 - p_2 e^{-d_{exon}/p_3} - p_4 e^{-d_{CNE}/p_5})$

C: $\pi/d \sim \exp[\log(p_1) - p_2 \sum_{i=1}^{n} e^{-x_i/p_3} - p_4 \sum_{i=1}^{m} e^{-x_i/p_5}]$

D: Background selection model with exponential distribution of heterozygous selection coefficients for exons with mean p_1 and CNEs with mean p_2.

p_1 .. p_5 are parameters estimated from the model, d_{exon} and d_{CNE} are the distance to the nearest exon and CNE respectively. In model C, summations are over *n* linked exonic sites and *m* linked CNE sites, where x_i measures the distance to a site (Materials and Methods). Distance in all models is either measured on a physical (bp) or genetic (cMs) scale. For ease of fitting models genetic distance in centiMorgans (cM) was scaled such that the magnitude of distances was comparable to that on a physical scale (measured in bp) by multiplying by a constant factor of 1,708,728 (Materials and Methods). r^2 is an estimate of the proportion of variance explained by the model, and ΔAIC is the Akaike information criterion relative to the best fitting model (for 200bp or 1000kb windows separately). Significance for parameter values, where appropriate, are provided in square brackets (*** = p<0.001).

Window Size (bp)	Scale	Model Label	p_1	p_2	p_3	p_4	p_5	r^2 (%)	ΔAIC
200	bp	A	3.77E-02 [***]	1.13E-03 [***]	7.67E-04 [***]	–	–	0.259	0
200	cM	A	3.75E-02 [***]	1.17E-03 [***]	7.72E-04 [***]	–	–	0.326	3389
200	bp	B	6.56E-02 [***]	1.31E-01 [***]	9.82E+03 [***]	9.86E-02 [***]	7.71E+02 [***]	0.300	2031
200	cM	B	6.73E-02 [***]	1.36E-01 [***]	1.18E+04 [***]	1.03E-01 [***]	1.09E+03 [***]	0.363	5213
200	bp	C	6.71E-02	4.14E-05	2.16E+04	2.01E-04	4.65E+03	0.511	–
200	cM	C	6.68E-02	4.57E-05	1.86E+04	2.07E-04	4.51E+03	0.497	–
200	bp	D	4E-5	2E-5	–	–	–	0.440	–
1000	bp	A	3.52E-02 [***]	1.12E-03 [***]	8.06E-04 [***]	–	–	1.15	0
1000	cM	A	3.52E-02 [***]	1.16E-03 [***]	8.00E-04 [***]	–	–	1.43	3129
1000	bp	B	6.30E-02 [***]	1.36E-01 [***]	9.54E+03 [***]	9.98E-02 [***]	6.26E+02 [***]	1.28	1389
1000	cMs	B	6.47E-02 [***]	1.41E-01 [***]	1.17E+04 [***]	1.01E-01 [***]	9.71E+02 [***]	1.54	4280
1000	bp	C	6.48E-02	4.10E-05	2.27E+04	2.28E-04	4.21E+03	2.18	–
1000	cMs	C	6.45E-02	4.70E-05	1.89E+04	2.40E-04	3.89E+03	2.14	–
1000	bp	D	4E-5	2E-5	–	–	–	1.88	–
