# Supplementary material for: Contributions of Protein-Coding and Regulatory Change to Adaptive Molecular Evolution in Murid Rodents
Source: PLoS Genet. 2013 Dec 5;9(12):e1003995. doi: 10.1371/journal.pgen.1003995 (PMC3854965; doi:10.1371/journal.pgen.1003995)
Supplement: Table S9 — Coverage statistics for sequences of 10 M. m. castaneus and one M. famulus individual. (PDF) [file pgen.1003995.s014.pdf]

Table S9. Coverage statistics for sequences of 10 *M. m. castaneus* and one *M. famulus* individual. All figures reported include aligned reads after removing duplicate reads.

Sample	H12	H14	H15	H24	H26	H27	H28	H30	H34	H36	***M. famulus***
Median coverage	22	35	27	28	27	32	28	44	22	29	25
Mean coverage	21	34	27	28	27	32	29	43	22	31	27
Covered > 0x	0.92	0.92	0.92	0.92	0.92	0.92	0.93	0.92	0.92	0.92	0.88
Covered > 10x	0.82	0.87	0.86	0.86	0.86	0.87	0.86	0.89	0.83	0.84	0.78
