# Supplementary material for: Contributions of Protein-Coding and Regulatory Change to Adaptive Molecular Evolution in Murid Rodents
Source: PLoS Genet. 2013 Dec 5;9(12):e1003995. doi: 10.1371/journal.pgen.1003995 (PMC3854965; doi:10.1371/journal.pgen.1003995)
Supplement: Text S1 — Additional details of methodology. (PDF) [file pgen.1003995.s015.pdf]

Supporting Information

Contributions of protein-coding and regulatory change to adaptive molecular evolution in murid rodents

Daniel L. Halligan[1], Athanasios Kousathanas[1], Rob W. Ness[1], Bettina Harr[2], Lél Eöry[3], Thomas M. Keane[4], David J. Adams[4] and Peter D. Keightley[1]

[1] *Institute of Evolutionary Biology, University of Edinburgh, West Mains Rd, Edinburgh, EH9 3JT, UK*

[2] *Max-Planck Institute for Evolutionary Biology, August-Thienemannstrasse 2, 24306 Plön, Germany*

[3] *The Roslin Institute and R(D)SVS, University of Edinburgh, Easter Bush, Midlothian EH25 9RG, UK.*

[4] *The Wellcome Trust Sanger Institute, Hinxton, Cambridge CB10 1HH, UK*

Estimation of the accuracy of Illumina sequencing. We attempted to check the accuracy of the Illumina SNP calls by comparing Illumina-based genotype calls with those made using traditional Sanger sequencing technology of the same individuals from a previous study [2]. We attempted to reduce the error rate within the Sanger based genotype calls by choosing regions of the genome for which we had Sanger sequence chromatograms in both directions, from coding sequence data only (where alignments are less error prone) and where both the forward and reverse sequence chromatograms were clear and had little background noise.

We were able to make genotype comparisons at a total of 16,249 sites, and were able to compare a total of 99,459 individual genotype calls. The majority of these sites were called as invariant by both methods, only 244 sites being called as variant by either method. At these sites, we observed a total of 55 discrepant SNP calls (over a total of 33 sites), however for 34 SNP calls (covering 20 sites) the error could confidently be assigned to the Sanger technology and for 19 SNP calls (11 sites) the error could tentatively be assigned to the Sanger technology. Assignment of the error to Sanger sequencing in these cases resulted from several observations. Firstly, in nine of these cases, we could identify strong evidence for the genotype called by Illumina in the Sanger chromatograms. In some cases, this was due to an incorrect heterozygous genotype code being used (e.g. R, implying A/G instead of Y implying C/T) when calling the Sanger genotypes, though it is worth noting that these errors would not affect the inferred site frequency spectrum. In other cases, where Sanger called a genotype as homozygous and Illumina called the genotype as heterozygous, it was clear upon re-inspection that two peaks were evident in the sequence chromatogram corresponding to the two bases called by Illumina. Secondly, in six cases, the Sanger and Illumina genotypes matched for all individuals, but genotypes for two individuals were swapped. The most parsimonious explanation for this would be an error in labelling tubes during preparation for Sanger sequencing, since this was only observed in two of the 80 amplicons. Again, an error of this type would not affect the inferred site frequency spectrum. Thirdly, for the remaining 19 cases, we could confidently assign the error to cases of single allele amplification when carrying out Sanger sequencing. In all of these cases, heterozygous individuals called by Illumina were homozygous when called by Sanger, the Sanger amplicons showed no heterozygosity throughout their length, and we could confidently identify a heterozygous position within one of the Sanger primer sites from the Illumina sequences of the individuals that were discrepant. Furthermore, in all of these cases the Illumina read depth was not abnormally high, which would be predicted if reads from paralogs were aligned to the same region.

For 19 discrepant SNP calls that could be tentatively assigned as Sanger errors, 13 were from a single Sanger amplicon. The SNP calls throughout this amplicon were consistent with three

individuals being swapped. The remaining six SNP calls tentatively assigned as Sanger errors were comprised of five homozygous genotype calls in Illumina, but heterozygous calls in Sanger and one heterozygous call in Illumina but homozygous in Sanger. In the first instance it is possible that background noise in sequence traces caused an incorrect genotype call in the Sanger technology. In the second instance the discrepancy could be due to a recent duplication combined with mapping of Illumina reads from a duplicate region to the same genomic section, or alternatively, single allele amplification of the Sanger amplicon.

In only one case could we confidently assign the error to Illumina sequencing (and in this case the reported genotype quality from SAMtools had an exceptionally low value of 3). In one other case we could tentatively assign the error to Illumina sequencing. The results indicate that for this dataset our Illumina sequencing is much more accurate than the Sanger sequence data for the same regions, and furthermore, that the Illumina sequencing based error rate is low. Accepting that we have two Illumina errors, the error rate = 2/99,459 = 0.002% per genotype call or 2/16,249 = 0.012% per site.

Inference of unfolded site frequency spectra to obtain M. m. castaneus lineage specific estimates of divergence. Inference of the unfolded site frequency spectrum (**u**, the number of sites with frequency i for $i = 1...n-1$ where n is the number of alleles sampled) can be problematic due to ancestral misidentification. In particular, some low-frequency derived variants can be incorrectly assigned as high-frequency derived variants if the ancestral state is incorrectly inferred, leading to an excess of high-frequency variants. This problem can occur if, for example, the ancestral state is inferred by parsimony and there are multiple hits between the ingroup and any outgroup sequences.

Recently, a method was developed to infer the ancestral state of a polymorphism (A) in a phylogeny containing an ingroup taxa segregating for two observed alleles (x and y) and two outgroup taxa (with observed alleles o_1 and o_2). The method calculates the probability that either x or y is ancestral for a set of segregating sites, and, using these, estimates the unfolded SFS (**u**) (Schneider et al. 2011). The method incorporates a general time reversible model of sequence evolution and also allows for rate variation amongst sites using a discrete approximation to the gamma distribution (Yang 2004). The likelihood of observing ancestral state $A = x$ is calculated as the product of the likelihood of observing a phylogeny with allele x at the tip of the in-group branch (summing over discrete rate variation classes and the possible states of the unknown internal node, Y) and the probability that ancestral state x generates a site segregating for x and y (given that the site is segregating).

$$\mathscr{L}(A=x|o_1,o_2,\theta)=\mathscr{L}(T|\theta,x,o_1,o_2).\,p(S=\{x,y\}|x,\theta),$$

where *θ* is the substitution process describing the substitution rate matrix **Q**, the branch lengths (**b**) and the gamma shape parameter (*a*) used to model rate variation amongst sites, and *T* is the tree relating the three taxa (Schneider et al. 2011). Assuming that the ancestral state *A* can only take states *x* and *y*, then the probabilities of observing the two possible
ancestral states can be obtained by normalising the likelihoods:

$$p(A=x)=\frac{\mathscr{L}(A=x|o_1,o_2,\theta)}{\mathscr{L}(A=x|o_1,o_2,\theta)+\mathscr{L}(A=y|o_1,o_2,\theta)}.$$

However, when calculating *p*(*A* = *x*) for a site, this method does not incorporate the observed frequency of alleles in the ingroup *x* and *y*, and as a result, can lead to biased inferences. Here, we incorporate this information by noting that the likelihood of observing the ancestral state *A* = *x*, given the state *x* of the major base, the observed states of the outgroups, the substitution process (*θ*) and the observed frequency of the major allele *x* (f_i), is:

$$\begin{aligned}\mathscr{L}(A=x|o_1,o_2,\theta,f_i) &= \mathscr{L}(A=x|o_1,o_2,\theta).\,p(f_i|A=x,S=\{x,y\}),\\ \mathscr{L}(A=y|o_1,o_2,\theta,f_i) &= \mathscr{L}(A=y|o_1,o_2,\theta).\,p(f_i|A=y,S=\{x,y\})\end{aligned}$$

where $p(f_i|A=x,S=\{x,y\})$ is the probability of observing major allele *x* at frequency f_i given that the site is segregating for *x* and *y* and *x* is ancestral and $p(f_i|A=y,S=\{x,y\})$ is the corresponding probability where allele *y* is ancestral. Note, however, that these probabilities correspond to elements of the unfolded SFS (**u**):

$$\begin{aligned}p(f_i|A=x,S=\{x,y\}) &= \frac{u_i}{S} = \frac{S_i d_i}{S},\\ p(f_i|A=y,S=\{x,y\}) &= \frac{u_{n-i}}{S} = \frac{S_i(1-d_i)}{S},\end{aligned}$$

for *i* in 1..(*n*/2), where *S* is the total number of sites, S_i is the number of sites where the major allele has frequency f_i and d_i is the probability that the major allele at frequency f_i is ancestral. As above, assuming that the ancestral state *A* is either *x* or *y*, then the probabilities of the two possible ancestral states (incorporating the observed allele frequency) an be obtained by normalising the likelihoods as above:

$$p(A=x) = \frac{\mathcal{L}(A=x|o_1,o_2,\theta,f_i)}{\mathcal{L}(A=x|o_1,o_{2,}\theta,f_i)+\mathcal{L}(A=y|o_1,o_2,\theta,f_i)}$$
$$= \frac{\mathcal{L}(A=x|o_1,o_2,\theta).d_i}{\mathcal{L}(A=x|o_1,o_2,\theta).d_i+\mathcal{L}(A=y|o_1,o_2,\theta).(1-d_i)}.$$

Although, d_i is unknown, it can be estimated using an expectation-maximisation algorithm. Starting with an initial guess for d_i (*e.g.* $d_i = 0.5$) an updated estimate of d_i can be obtained as the expected value of $p(A = x)$ over sites whose major allele frequency is f_i, which can be iterated until convergence:

$$d_i=\frac{1}{S_i}\sum_{j=1}^{S_i} p(A=x)_j,$$

u can then be estimated from the folded SFS (**u'**) using estimates of **d** for each value of $i = 1...n$:

For our data we inferred the unfolded SFS for sites with 20 alleles, we therefore needed to estimate d_i values from $i = 0...9$.

$$u_i=\begin{cases} u'_i d_i & \text{if } i<n/2; \\ u'_{(n-i)}(1-d_i) & \text{if } i>n/2. \end{cases}$$

Checks on estimates of α and ωa. The calculations of α and ω_a presented in the main text make a number of assumptions, the robustness of which we investigated in several ways. Firstly, we obtained estimates of α and ω_a using divergence calculated between *M. m. castaneus* and *M. famulus*, or the *M. m. castaneus* lineage-specific divergence, estimated using *M. famulus* and rat as outgroups to infer down the *M. m. castaneus* lineage since the split with *M. famulus*. Estimates of α and ω_a obtained are similar to those estimated from divergence with rat (Table S2) and provide support that our estimates of the rate of adaptation are not strongly affected by long-term population size change down the mouse or rat lineages. The somewhat higher estimates of α and ω_a based on *M. famulus* as an outgroup compared to those based on rat as an outgroup may be a consequence of ancestral polymorphism contributing to divergence [52] or a lower effective population size in *M. famulus* since the split with *M. m. castaneus*. CNEs have higher estimates of α and ω_a when using *M. famulus* as an outgroup, which may represent a relatively bigger contribution of ancestral polymorphism to apparent divergence in CNEs *vs.* exons.

The neutral standard used for CNEs is not interdigitated with the selected sites we use, but instead is chosen to be 500bp upstream/downstream of the region identified as being conserved

(Supplementary Methods). Recombination events between these flanking neutral standard and the selected sequences can result in bias in estimates of α and ω_a [22]. Therefore, in order to check the validity of our neutral standard sequence, we obtained estimates of α and ω_a using sections of DNA at different offsets from the CNEs (i.e., both 200bp and 1,000bp removed from the CNEs). However, estimates of α and ω_a inferred in CNEs are relatively insensitive to the location of putatively neutral control sequences (Table S3, S5).

Another potential factor that may bias estimates of α and ω_a within CNEs is that many CNEs are located close to genic sequences and, as a result, diversity within CNEs and their flanks may be reduced below that expected for neutrality due to linked selection on exons. This effect could potentially bias estimates of α and ω_a, if there is a differential effect of CNEs and their flanks, e.g. due to distance from the exons. We tested this effect by defining two categories of CNEs: proximal CNEs (pCNEs, within 20Kb of any known exon) and distal CNEs (dCNEs, more than 20Kb from any known exon).

Verifying the extent of π/d reductions in exon and CNE flanks. It is possible that the reductions in π/d observed in exon and CNE flanks could be obscured by direct selection operating on non-exonic or non-CNE sequences located in exon and CNE flanks. To investigate this possibility, we analysed subsets of flanking sequences where we attempted to remove any effects of direct negative selection. For the flanks of CNEs, we attempted to remove the effects of direct negative selection firstly by excluding sites immediately flanking CNEs, which show substantially reduced divergence between mouse and rat, consistent with direct negative selection (Figure 2). Secondly, we identified the location of remnants of transposable elements inserted prior to the split of mouse and rat (ancestral repeats), which appear to be a good candidate for neutrally evolving sequences in mammals [26] and only included sites from the flanks that were identified as belonging to an ancestral repeat. Thirdly, we examined patterns of π/d surrounding CNEs located far from exons (dCNEs), which should be less influenced by the effects of selection acting on exonic sequences and surrounding CNEs that were identified from multiple alignments that include mouse and rat (mCNEs). To attempt to remove the effects of direct selection from the flanks of exons, firstly, we only analysed sites from ancestral repeats in the flanks of exons. Secondly, we excluded any sites in the immediate flanks (adjacent 500bp) of CNEs from contributing to the data from exon flanks. We quantified the depth and extent of the reductions in π/d observed for the subsets of the sequences flanking exons and CNEs by fitting the simple exponential model as described above.

Estimates of the width and depth of the reductions in *π/d* analysing subsets of sites in the flanks of exons and CNEs are reported in Table S7. These estimates are similar when only examining sites flanking CNEs or exons that are also within ancestral repeats and when we exclude sites immediately adjacent to CNEs (either 500bp of 1,000bp, which show reduced divergence on average and may therefore be subject to negative selection, Figure 2). The estimates are also similar if we only consider the flanks of CNEs located far from exons. When we define CNEs from multiple alignments that include mouse and rat estimates are comparable, though the estimated depth of the trough in diversity around CNEs is slightly (1.16x) larger, indicating that the results are largely unaffected by our requirement that the CNEs must have an identifiable orthologous sequence in humans. Similarly, our estimates of the depth and width of depressions in *π/d* in the flanks of exons are quantitatively similar if we only analyse ancestral repeats located in the flanks of exons and if we exclude the immediate 500bp flanks of CNEs (Table S7).

Modelling relative diversity within non-overlapping windows in the genome. We attempted to model *π/d* in non-overlapping windows around the genome using a range of models. Initially, we fitted a model where *π/d* was a linear combination of log distance to the nearest exon and nearest CNE (model A). Under this model, both log distance to the nearest exon and CNE have a significant effect on *π/d* (this is true whether we calculate *π/d* in 200bp or 1Kb windows). This implies that reductions in *π/d* can be attributed to both categories of element. So, for example, the model suggests that the reductions in diversity observed in exon flanks are due to the presence of the exons themselves, rather than being due to CNEs that are clustered near exons. Interestingly, if we fit the model using genetic instead of physical distance, we obtain an improved fit (i.e., a greater proportion of variance in *π/d* can be explained as measured by r^2).

We then attempted to fit a more complex non-linear model where *π/d* is modelled as a exponential function of distance to the nearest exon and nearest CNE (model B). This allows us to estimate the relative reductions in *π/d* attributable to the nearest exon or CNE and the distance over which these reductions extend. Consistent with the patterns observed in Figure 1, the results from this model imply that *π/d* is reduced by a similar amount in the immediate flanks of exons and CNEs (by ~13% and ~10% respectively on both a physical and genetic distance scale) and that the width of this reduction is approximately an order of magnitude larger for exons than CNEs (Table S8). Again, this result suggests that both exons and CNEs are associated with reductions in *π/d*.

Additional References for Supporting Text

Schneider, A., Charlesworth, B., Eyre-Walker, A. and Keightley, P. D. (2011). A method for inferring the rate of occurrence and fitness effects of advantageous mutations. *Genetics* 189: 1427-1437.

Yang, Z., 1994 Maximum likelihood phylogenetic estimation from DNA sequences with variable rates over sites: approximate methods. J. Mol. Evol. 39: 306–314

Table S1. Demographic parameter estimates for 2-epoch demographic model and differences in log likelihood between 2-epoch and constant population (*ΔlogL*) model fitted to folded SFSs for putatively neutral classes of sites when analysing non-CpG-prone sites.

Site Class	N_2/N_1	t_2/N_1	*ΔlogL*
Synonymous	2.79<br>[2.79,3.07]	1.47<br>[1.33,2.13]	1719
CNE neutral reference	2.79<br>[2.79,2.79]	1.61<br>[1.55,1.69]	32321
pCNE neutral reference	2.79<br>[2.79,2.79]	1.76<br>[1.63,1.88]	11798
dCNE neutral reference	2.79<br>[2.79,2.79]	1.55<br>[1.47,1.63]	20536

Table S2. Estimates of selection parameters from a DFE-alpha analysis of folded SFSs for non-CpG-prone sites for different site classes.

Site Class	$N_w s_{del}$	β	α (cas)	ω_a (cas)	α (fam)	ω_a (fam)	α (rat)	ω_a (rat)
Zero-fold	9.5e5 [3.5e7, 2.6e5]	0.11 [0.088, 0.13]	0.2 [0.16, 0.24]	0.042 [0.032, 0.049]	0.32 [0.29, 0.37]	0.075 [0.068, 0.095]	0.32 [0.28, 0.35]	0.077 [0.067, 0.087]
Two-fold (nonsyn.)	→∞ [→∞, 4.0e9]	→0 [→0, 0.066]	0.19 [0.15, 0.24]	0.046 [0.036, 0.059]	0.31 [0.29, 0.38]	0.089 [0.082, 0.12]	0.38 [0.36, 0.42]	0.12 [0.12, 0.14]
UTRs	250 [500, 140]	0.050 [0.050, 0.050]	0.039 [-0.0053, 0.083]	0.026 [-0.0035, 0.057]	0.21 [0.18, 0.28]	0.17 [0.15, 0.25]	0.19 [0.16, 0.23]	0.15 [0.12, 0.19]
CNEs	45 [50, 40]	0.16 [0.15, 0.17]	0.25 [0.23, 0.26]	0.13 [0.12, 0.14]	0.37 [0.36, 0.39]	0.24 [0.23, 0.25]	0.19 [0.18, 0.21]	0.097 [0.091, 0.10]
pCNE	81 [110, 63]	0.12 [0.11, 0.13]	0.23 [0.21, 0.25]	0.13 [0.12, 0.15]	0.35 [0.32, 0.37]	0.24 [0.22, 0.26]	0.18 [0.16, 0.19]	0.097 [0.086, 0.11]
dCNE	31 [34, 28]	0.19 [0.19, 0.20]	0.25 [0.24, 0.27]	0.12 [0.12, 0.13]	0.39 [0.37, 0.40]	0.23 [0.22, 0.24]	0.20 [0.19, 0.22]	0.094 [0.088, 0.10]

$N_w s_{del}$ is the scaled mean effect of a deleterious mutation. β is the shape parameter of gamma distribution. Estimates of α and ω_a are provided for three possible divergences: the *M. m. castaneus* only branch (using the inferred number of fixed differences from the unfolded SFS), *M. m. castaneus-M. famulus* (using mean divergence between *M. m. castaneus* and *M. famulus*) and *M. m. castaneus*-rat (using mean divergence between *M. m. castaneus* and rat). Note that in all cases, divergence is corrected for multiple hits using a Jukes-Cantor correction, and for *M. m. castaneus-M. famulus and M. m. castaneus*-rat we also correct divergence for the potential contribution of polymorphism [52] pCNEs and dCNEs are defined as CNEs located less than 20Kb and more than 20Kb from an annotated exon respectively. 95% bootstrap confidence intervals, shown in square brackets, were calculated by bootstrapping 1,000 times by gene (in the case of zero-fold and two-fold nonsynonymous sites and UTRs) or by 10,000bp sections of the genome (in the case of CNEs, pCNEs and dCNEs).

Table S3. Estimated DFE parameters and rates of adaptive molecular evolution obtained when using ancestral repeats located within introns (excluding sites that may function as intronic splice sites, defined as the first and last 30bp of each intron) as a neutral standard.

Site Class	$N_w s_{del}$	β	**α (cas)**	**ω_a (cas)**	**α (fam)**	**ω_a (fam)**	**α (rat)**	**ω_a (rat)**
Zero-fold	3.1e4	0.17 [0.16, 0.18]	0.46 [0.44, 0.48]	0.11 [0.10, 0.11]	0.56 [0.53, 0.58]	0.16 [0.15, 0.16]	0.45 [0.42, 0.27]	0.10 [0.092, 0.11]
Two-fold (nonsyn.)	5.4e6 [9.3e8, 3.4e5]	0.11 [0.079, 0.13]	0.45 [0.41, 0.49]	0.12 [0.11, 0.4]	0.55 [0.51, 0.58]	0.18 [0.17, 0.20]	0.49 [0.46, 0.53]	0.15 [0.14, 0.16]
UTRs	5.4e3 [9.8e3, 3.1e3]	0.05 [0.05, 0.05]	0.27 [0.24, 0.30]	0.20 [0.18, 0.23]	0.40 [0.37, 0.42]	0.37 [0.33, 0.40]	0.25 [0.23, 0.28]	[0.19 [0.17, 0.21]
CNEs	25 [27,24]	0.23 [0.22, 0.24]	0.39 [0.38, 0.40]	0.21 [0.20, 0.22]	0.51 [0.50, 0.52]	0.35 [0.34, 0.36]	0.32 [0.31, 0.33]	0.15 [0.15, 0.16]

Table S4. Changes in log likelihood (ΔlogL) between 2-epoch and 3-epoch demographic models and parameter estimates for 3-epoch model.

Site class	ΔlogL	N_2	t_2	N_3	t_3
Four-fold	7.0	2	7.4	50	29
CNE neutral reference	45.1	40	542	110	52

Table S5. Demographic and selection parameter estimates for pCNEs and dCNEs using putatively neutrally evolving standard sequences offset from the CNEs by 200, 500 and 1000bp. Estimates are obtained for non-CpG-prone sites only using rat as an outgroup.

Site Class	**Neutral reference offset (bp)**	N_2/N_1	t_2/N_1	$N_e s$	β	α **(rat)**	ω_a **(rat)**
dCNE	200	3.07	1.55	-39.9	0.153	0.150	0.0742
dCNE	500	2.79	1.55	-30.8	0.194	0.204	0.0937
dCNE	1000	2.79	1.77	-32.2	0.204	0.230	0.104
pCNE	200	2.79	1.33	-103	0.101	0.139	0.0796
pCNE	500	2.79	1.76	-81.4	0.120	0.177	0.0968
pCNE	1000	2.79	1.88	-77.6	0.126	0.184	0.0991

Table S6. Estimated parameters of a DFE model with three discrete mutation fitness effect bins (s=0, s=s_2 and s=1) with proportions (p_1, p_2 and p_3), the average fitness effect (weighted average of 0, s_2 and 1), the change in log likelihood from a gamma model of the DFE ($\Delta \log L = \log L_{discrete} - \log L_{gamma}$), the mean fixation probability (u_n) and estimates of α and ω_a. Estimates are obtained for non-CpG-prone sites only using rat as an outgroup.

Site Class	$N_e s_2$	p_1	p_2	p_3	***Mean*** $N_e s$	$\Delta \log L$	u_N	α **(rat)**	ω_a **(rat)**
Zero-fold	7.63	0.189	0.0276	0.786	143	18.7	0.187	0.224	0.053
Two-fold (nonsyn.)	27.7	0.209	0.0	0.791	144	-6.42	0.209	0.353	0.114
UTRs	2.47	0.72	0.0	0.282	51	-4.82	0.718	0.106	0.0851
CNEs	7.20	0.46	0.287	0.249	48.6	57.9	0.464	0.0677	0.0336

Table S7. Estimated reductions in diversity in the flanks of exons and CNEs. To quantify reductions in diversity we fitted the function $f(x)=A(1-B.\exp(-x/d))$ to mean π/d calculated for across non-overlapping windows flanking exons and CNEs separately by non-linear least squares. In all cases we excluded CNEs from the flanks of exons and any annotated exons from the flanks of CNEs. The categories are defined as follows:

1. CNEs: CNE flanks
2. CNEs (AR only): CNEs flanks restricted to ancestral repeats only
3. CNEs (exc. adjacent 500): CNEs flanks excluding the 500bp adjacent to each CNE excluded
4. CNEs (exc. adjacent 1,000): CNEs flanks excluding the 1,000bp adjacent to each CNE excluded
5. dCNEs: Flanking sequences of dCNEs only (CNEs located >20Kb from any exon)
6. mCNEs: Flanking sequences of mCNEs (see Supplementary Methods for description)
7. Exons: Exon flanks
8. Exons (AR only): Exon flanks restricted to ancestral repeats only
9. Exons (exc. CNE flanks): Exon flanks excluding not only CNEs, but also 500bp upstream and downstream of every CNE.

Site Class	***A***	***d***	***Width (d.ln2)***	***Depth (B)***
CNEs	0.0501	1.17	0.809	0.108
CNEs (AR only)	0.0507	1.19	0.827	0.0675
CNEs (exc. adjacent 500bp)	0.0501	1.19	0.825	0.107
CNEs (exc. Adjacent 1,000bp)	0.0501	1.24	0.859	0.103
dCNEs	0.0507	1.21	0.838	0.0671
mCNEs	0.0499	1.21	0.836	0.125
Exons	0.0511	13.0	9.04	0.152
Exons (AR only)	0.0530	12.5	8.65	0.156
Exons (exc. CNE flanks)	0.0513	10.6	7.35	0.150

Table S8. Parameter estimates of models to of *π*/*d* calculated in 200bp for 1000bp non-overlapping windows around the genome. The models are:

A:π/d∼p1+p2log(dexon)+p3log(dCNE)

B:π/d∼1(1−2e−dexon/p3−p4e−dCNE/p5)

C:π/d∼exp[log(p1)−2∑i=1ne−xi/p3−p4∑i=1me−xi/p5]

D: Background selection model with exponential distribution of heterozygous selection coefficients for exons with mean p_1 and CNEs with mean p_2.

p_1 .. p_5 are parameters estimated from the model, d_{exon} and d_{CNE} are the distance to the nearest exon and CNE respectively. In model C, summations are over *n* linked exonic sites and *m* linked CNE sites, where x_i measures the distance to a site (Materials and Methods). Distance in all models is either measured on a physical (bp) or genetic (cMs) scale. For ease of fitting models genetic distance in centiMorgans (cM) was scaled such that the magnitude of distances was comparable to that on a physical scale (measured in bp) by multiplying by a constant factor of 1,708,728 (Materials and Methods). r^2 is an estimate of the proportion of variance explained by the model, and ΔAIC is the Akaike information criterion relative to the best fitting model (for 200bp or 1000kb windows separately). Significance for parameter values, where appropriate, are provided in square brackets (*** = p<0.001).

Window Size (bp)	Scale	Model Label	p_1	p_2	p_3	p_4	p_5	r^2 (%)	ΔAIC
200	bp	A	3.77E-02 [***]	1.13E-03 [***]	7.67E-04 [***]	–	–	0.259	0
200	cM	A	3.75E-02 [***]	1.17E-03 [***]	7.72E-04 [***]	–	–	0.326	3389
200	bp	B	6.56E-02 [***]	1.31E-01 [***]	9.82E+03 [***]	9.86E-02 [***]	7.71E+02 [***]	0.300	2031
200	cM	B	6.73E-02 [***]	1.36E-01 [***]	1.18E+04 [***]	1.03E-01 [***]	1.09E+03 [***]	0.363	5213
200	bp	C	6.71E-02	4.14E-05	2.16E+04	2.01E-04	4.65E+03	0.511	–
200	cM	C	6.68E-02	4.57E-05	1.86E+04	2.07E-04	4.51E+03	0.497	–
200	bp	D	4E-5	2E-5	–	–	–	0.440	–
1000	bp	A	3.52E-02 [***]	1.12E-03 [***]	8.06E-04 [***]	–	–	1.15	0
1000	cM	A	3.52E-02 [***]	1.16E-03 [***]	8.00E-04 [***]	–	–	1.43	3129
1000	bp	B	6.30E-02 [***]	1.36E-01 [***]	9.54E+03 [***]	9.98E-02 [***]	6.26E+02 [***]	1.28	1389
1000	cMs	B	6.47E-02 [***]	1.41E-01 [***]	1.17E+04 [***]	1.01E-01 [***]	9.71E+02 [***]	1.54	4280
1000	bp	C	6.48E-02	4.10E-05	2.27E+04	2.28E-04	4.21E+03	2.18	–
1000	cMs	C	6.45E-02	4.70E-05	1.89E+04	2.40E-04	3.89E+03	2.14	–
1000	bp	D	4E-5	2E-5	–	–	–	1.88	–

Table S9. Coverage statistics for sequences of 10 *M. m. castaneus* and one *M. famulus* individual. All figures reported include aligned reads after removing duplicate reads.

Sample	H12	H14	H15	H24	H26	H27	H28	H30	H34	H36	*M. famulus*
Median coverage	22	35	27	28	27	32	28	44	22	29	25
Mean coverage	21	34	27	28	27	32	29	43	22	31	27
Covered > 0x	0.92	0.92	0.92	0.92	0.92	0.92	0.93	0.92	0.92	0.92	0.88
Covered > 10x	0.82	0.87	0.86	0.86	0.86	0.87	0.86	0.89	0.83	0.84	0.78
